# Publisher Correction: scDesign2: a transparent simulator that generates high-fidelity single-cell gene expression count data with gene correlations captured

**DOI:** 10.1186/s13059-021-02394-z

**Published:** 2021-06-09

**Authors:** Tianyi Sun, Dongyuan Song, Wei Vivian Li, Jingyi Jessica Li

**Affiliations:** 1 Department of Statistics, University of California, Los Angeles, CA 90095-1554 USA; 2 Interdepartmental Program of Bioinformatics, University of California, Los Angeles, CA 90095-7246 USA; 3 Department of Biostatistics and Epidemiology, Rutgers School of Public Health, Piscataway, NJ 08854 USA; 4 Department of Human Genetics, University of California, Los Angeles, CA 90095-7088 USA; 5 Department of Computational Medicine, University of California, Los Angeles, CA 90095-1766 USA; 6 Department of Biostatistics, University of California, Los Angeles, CA 90095-1772 USA

**Correction to: Genome Biol 22, 163 (2021)**

**https://doi.org/10.1186/s13059-021-02367-2**

Following publication of the original paper [1], it was noticed that a typesetting error occurred.

The HTML version of this article erroneously contained the incorrect version of Table 1. It was also reported that “SSPsimSeq” should be “SPsimSeq” in the 12th row of Table 1. The correct Table 1 is given below.

**Table 1:** Summary of 16 simulators (including our proposed scDesign2) in six properties

Property	1 protocol adaptive	2 gene preserved	3 gene cor. captured	4 cell num. seq. dep. flexible	5 transparent	6 comp. and sample efficient
**Simulator**						
dyngen [77]		✗	✗		✓	✓
Lun2 [78]		✓	✗	✓	✓	✓
powsimR [75]	✓	✓	✗	✓	✓	✓
PROSST [68]		✓	✗		✓	✓
scDD [74]	✓	✗	✗		✓	✓
scDesign[35]	✓		✗	✓	✓	✓
scGAN [72]	✓	✓			✗	✗
splat simple[69]	✓	✗	✗	✗	✓	✓
splat [69]	✓	✗	✗	✗	✓	✓
kersplat [69]	✓	✗		✗	✓	✓
SPARSim [71]	✓	✓		✗	✓	✓
SPsimSeq [79]	✓	✓	✓	^**^	✓	✓
SymSim[70]	✓	✗	✗	✗	✓	✓
ZINB-WaVe[39]	✓			✗	✓	✓
SERGIO [76]	✓		✗*	✓	✓	✓
**scDesign2**	✓	✓	✓	✓	✓	✓

Property 1: protocol adaptiveness

Property 2: gene preservation

Property 3: gene correlation capture

Property 4: flexible cell number and sequencing depth choices

Property 5: transparency

Property 6: computational and sample efficiency

For each simulator and each property, a checkmark, checkcross, or cross means that the simulator satisfies, partially satisfies, or does not satisfy the property, respectively

^*^SERGIO requires a user-specified gene regulatory network, and it does not capture/estimate gene correlations from a real dataset

^**^SPsimSeq can vary cell number but not sequencing depth

The original article [1] has been updated.
