# Author Correction: scDesign2: a transparent simulator that generates high-fidelity single-cell gene expression count data with gene correlations captured

**DOI:** 10.1186/s13059-023-02884-2

**Published:** 2023-02-22

**Authors:** Tianyi Sun, Dongyuan Song, Wei Vivian Li, Jingyi Jessica Li

**Affiliations:** 1 Department of Statistics, University of California, 90095-1554 Los Angeles, CA USA; 2 Interdepartmental Program of Bioinformatics, University of California, 90095-7246 Los Angeles, CA USA; 3 Department of Biostatistics and Epidemiology, Rutgers School of Public Health, 08854 Piscataway, NJ USA; 4 Department of Human Genetics, University of California, 90095-7088 Los Angeles, CA USA; 5 Department of Computational Medicine, University of California, 90095-1766 Los Angeles, CA USA; 6 Department of Biostatistics, University of California, 90095-1772 Los Angeles, CA USA

Following the publication of the original paper [1], the authors identified two errors in the notation and formulas in the Methods section.
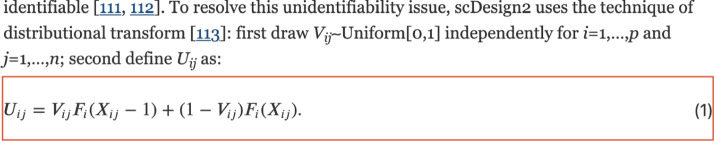


Equation (1) in the online version.

The first error is the $U_{ij}$ expression. Specifically, Eq. (1) should be corrected as

1 $$\begin{aligned} U_{ij}=(1-V_{ij})F_i(X_{ij}-1)+V_{ij}F_i(X_{ij})\,. \end{aligned}$$

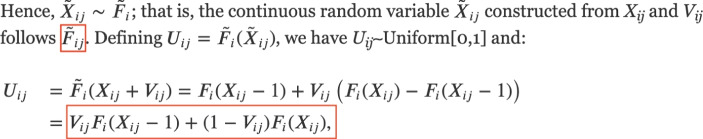


The end of the derivation of Eq. (1) in the online version.

Accordingly, at the end of the derivation of

$$\begin{aligned} U_{ij}= & {} \tilde{F}_i(X_{ij} + V_{ij}) = F_i(X_{ij} - 1) + V_{ij}\left( F_i(X_{ij}) - F_i(X_{ij}-1)\right) \\= & {} V_{ij}F_i(X_{ij}-1)+(1-V_{ij})F_i(X_{ij})\, , \end{aligned}$$

The last line should be changed to

$$\begin{aligned} U_{ij}= & {} \cdots \\= & {} (1-V_{ij})F_i(X_{ij}-1)+V_{ij}F_i(X_{ij})\,, \end{aligned}$$

The second error is one notation in the sentence just before the above-corrected expression. In “Hence, $\tilde{X}_{ij} \sim \tilde{F}_i$; that is, the continuous random variable $\tilde{X}_{ij}$ constructed from $X_{ij}$ and $V_{ij}$ follows $\tilde{F}_{ij}$”, at the end, $\tilde{F}_{ij}$ should be corrected as $\tilde{F}_{i}$.

The authors confirm that the notational errors corrected here had no effect on the conclusions or the performance of the method scDesign2.
