# Supplementary material for: scDesign2: a transparent simulator that generates high-fidelity single-cell gene expression count data with gene correlations captured
Source: Genome Biol. 2021 May 25;22:163. doi: 10.1186/s13059-021-02367-2 (PMC8147071; doi:10.1186/s13059-021-02367-2)
Supplement: Supplementary file 2 — Additional file 2 Comparison of scDesign2 to SPsimSeq. [file 13059_2021_2367_MOESM2_ESM.pdf]

Comparison of scDesign2 to SPsimSeq

To compare the quality of the synthetic data of scDesign2 and SPsimSeq, we perform two sets of benchmark analyses on the four scRNA-seq datasets and the MERFISH dataset, which are used in the comparisons with the other simulators in the main text.

First, we select the same three cell types from each scRNA-seq dataset (measured by each of the four experimental protocols), obtaining the same 12 cell-type–protocol combinations. Then for each combination, we compare the gene correlation matrix estimated from the test data with the matrices estimated from the synthetic data generated by scDesign2 and SPsimSeq; the comparison is made for the 100 genes with the highest mean expression values in the test data, and we compare both the Pearson correlation matrices and the Kendall's tau matrices. To quantify the difference between two matrices, one from the test data and one from the synthetic data, we compute the mean squared error (MSE), the smaller the better; to quantify the similarity between two matrices, we compute the Pearson correlation coefficient (PCor), the larger the better (for the calculation of PCor, we have excluded the diagonal entries, which are all ones, from each correlation matrix). In summary, a smaller MSE and a larger PCor indicate better resemblance of the synthetic data to the test data in terms of gene correlations. The results are summarized in Figures A1–A4: compared to SPsimSeq, scDesign2 achieves lower MSEs in 10 out of the 12 cell-type–protocol combinations, and higher PCors in 10 out of the 12 combinations (note that the 10 combinations are not all the same for the two criteria). This demonstrates that scDesign2 captures gene correlations more accurately than SPsimSeq does.

Second, for each of the four scRNA-seq datasets and the MERFISH dataset, we use 2D visualization—t-SNE and PCA—to compare cells of multiple types in the test data and the synthetic data generated by each simulator (Figures A5–A9). Both t-SNE and PCA 2D plots show that the synthetic cells generated by scDesign2 resemble the cells in test data more than the synthetic cells generated by SpsimSeq do. In particular, by overlaying real and synthetic cells in the same 2D plot, we find that the synthetic cells generated by scDesign2 are less distinguishable from real cells. This is indicated by the miLISI values, which are consistently higher for scDesign2 in both the t-SNE and PCA overlaying plots across all five datasets. This again demonstrates that scDesign2 outperforms SPsimSeq in generating realistic synthetic data.

Additional Figures

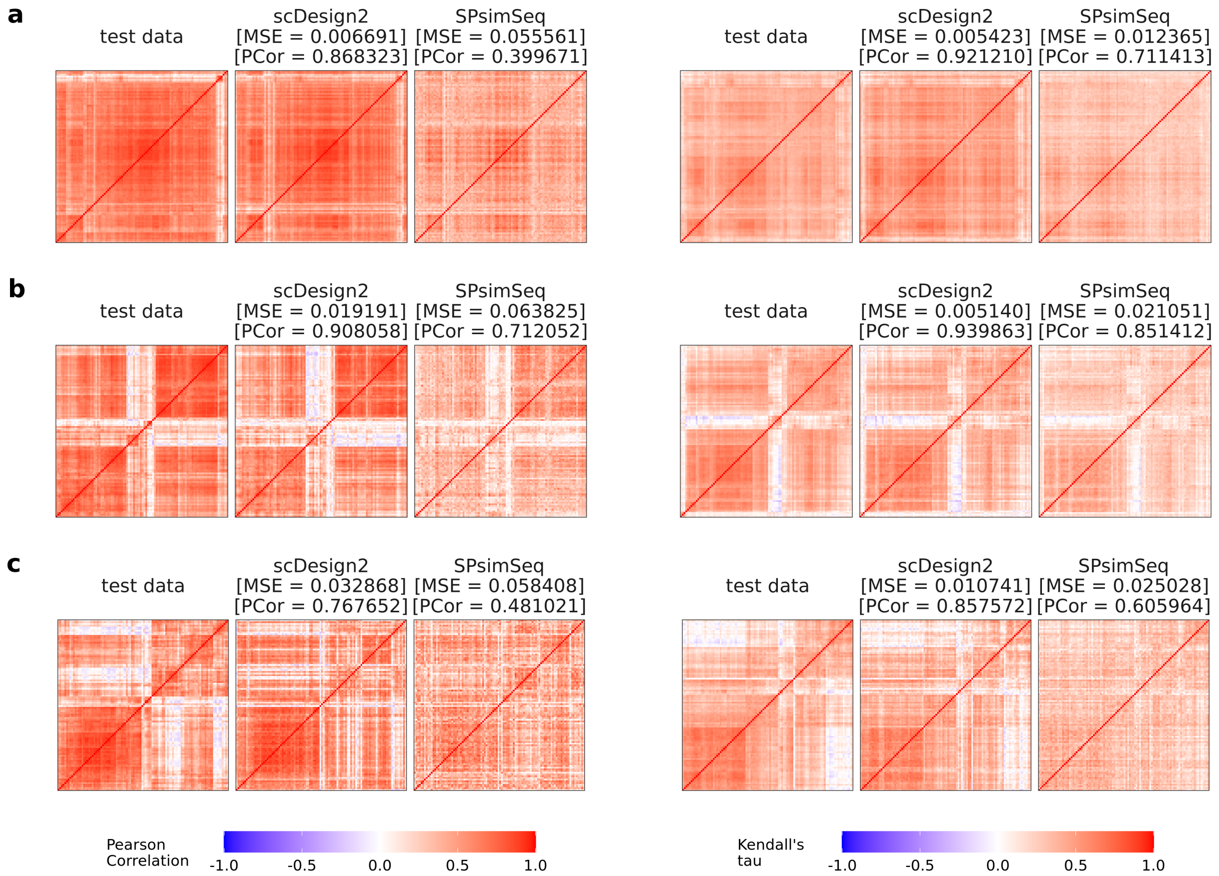

Figure A1: Heatmaps of gene correlation matrices estimated from the test data (real data) and synthetic data generated by scDesign2 and SPsimSeq. The comparison is performed for three cell types from the 10x Genomics dataset. The three cell types are (a) stem cells, (b) goblet cells, and (c) tuft cells. For each cell type, the Pearson correlation matrices (left column) and Kendall's tau matrices (right column) are shown for the 100 genes with the highest mean expression values in the test data; the rows and columns (i.e., genes) of all the matrices are ordered by the complete-linkage hierarchical clustering of genes (using Pearson correlation as the similarity in the left column and Kendall's tau in the right column) in the test data. We also calculate the mean squared error (MSE) and the Pearson correlation coefficient (PCor) between the values of each correlation matrix of the synthetic data and the corresponding correlation matrix of the test data. For the calculation of PCor, we have excluded the diagonal values of ones in the correlation matrices. We can see that in all the comparisons, scDesign2 achieves lower MSEs and higher PCors than SPsimSeq does.

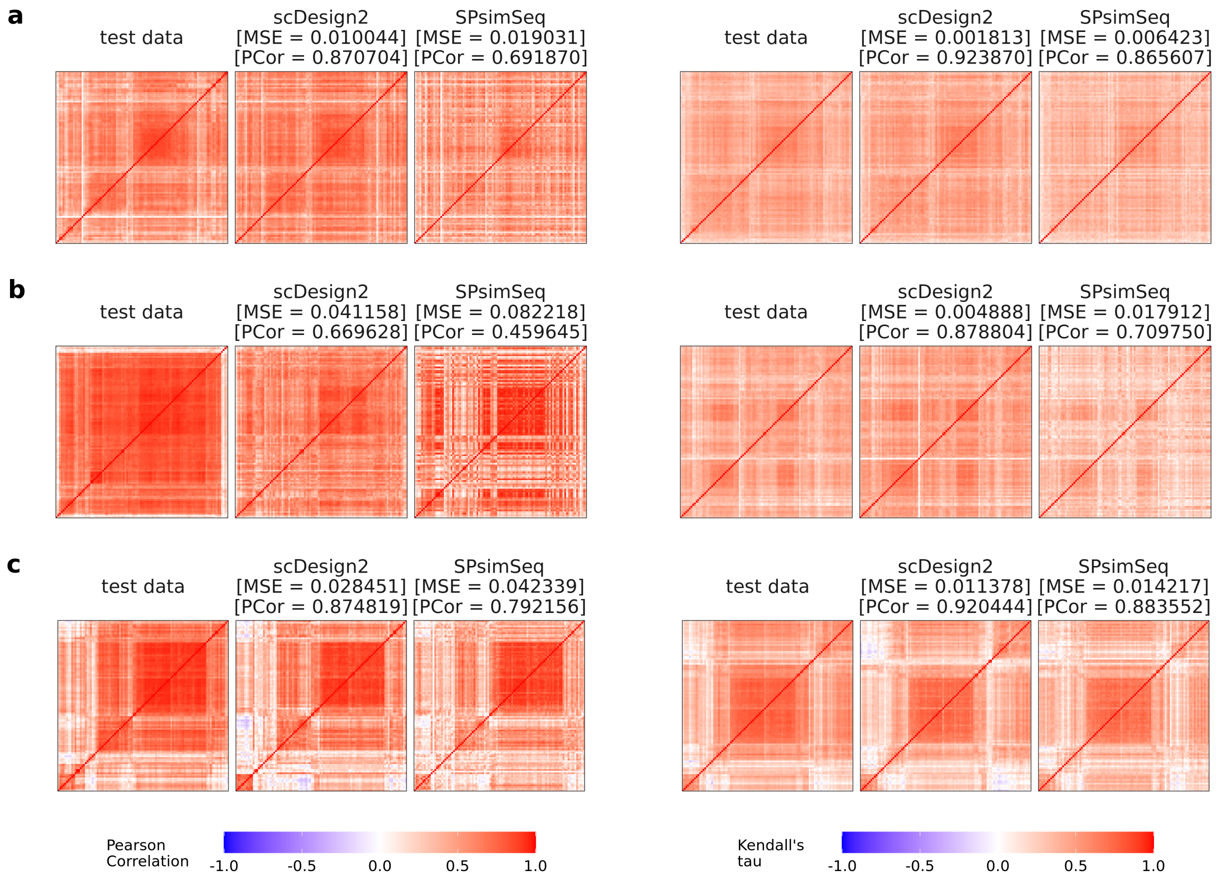

Figure A2: Heatmaps of gene correlation matrices estimated from the test data (real data) and synthetic data generated by scDesign2 and SPsimSeq. The comparison is performed for three cell types from the CEL-Seq2 dataset. The three cell types are (a) alpha cells, (b) beta cells, and (c) acinar cells. For each cell type, the Pearson correlation matrices (left column) and Kendall's tau matrices (right column) are shown for the 100 genes with the highest mean expression values in the test data; the rows and columns (i.e., genes) of all the matrices are ordered by the complete-linkage hierarchical clustering of genes (using Pearson correlation as the similarity in the left column and Kendall's tau in the right column) in the test data. We also calculate the mean squared error (MSE) and the Pearson correlation coefficient (PCor) between the values of each correlation matrix of the synthetic data and the corresponding correlation matrix of the test data. For the calculation of PCor, we have excluded the diagonal values of ones in the correlation matrices. We can see that in all the comparisons, scDesign2 achieves a lower MSE and a higher PCor than SPsimSeq.

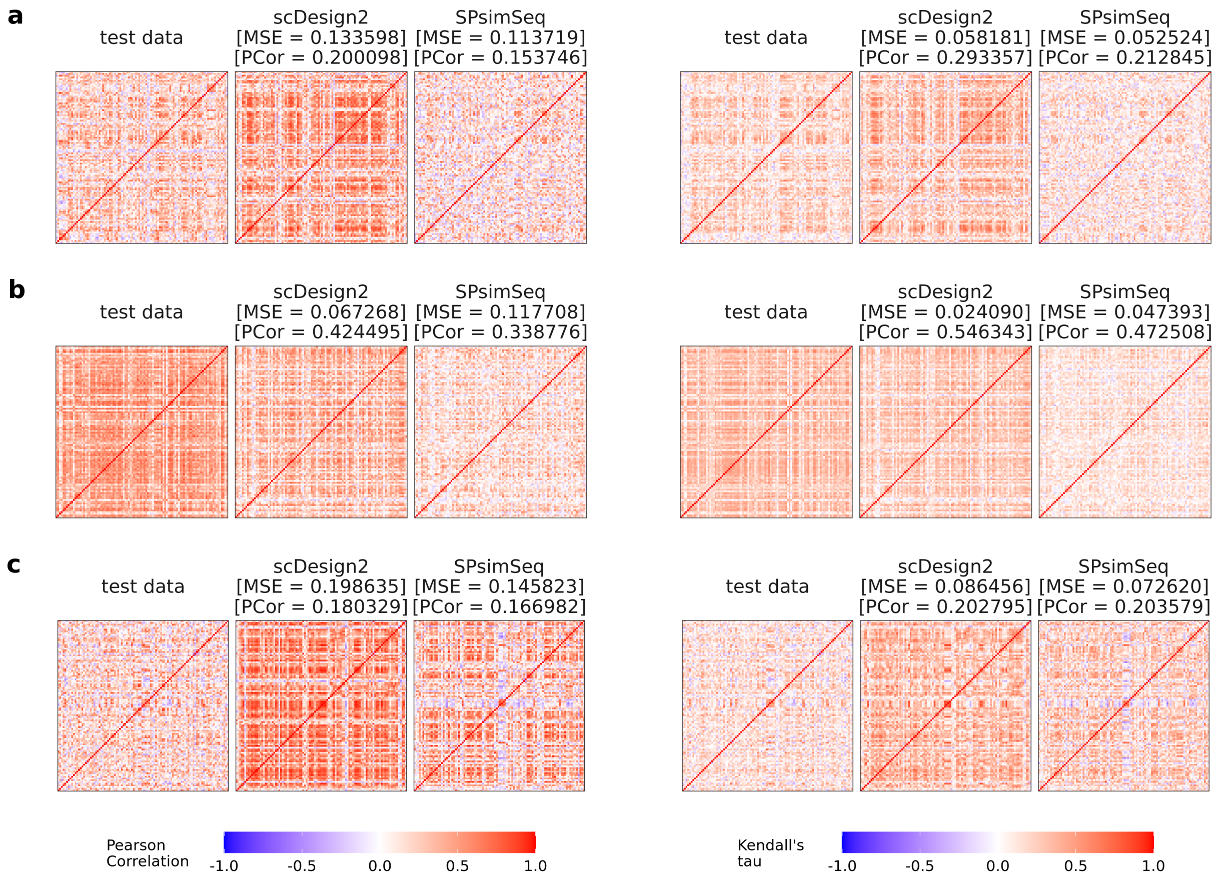

Figure A3: Heatmaps of gene correlation matrices estimated from the test data (real data) and synthetic data generated by scDesign2 and SPsimSeq. The comparison is performed for three cell types from the Fluidigm C1 dataset. The three cell types are (a) astrocytes, (b) neurons, and (c) oligodendrocytes. For each cell type, the Pearson correlation matrices (left column) and Kendall's tau matrices (right column) are shown for the 100 genes with the highest mean expression values in the test data; the rows and columns (i.e., genes) of all the matrices are ordered by the complete-linkage hierarchical clustering of genes (using Pearson correlation as the similarity in the left column and Kendall's tau in the right column) in the test data. We also calculate the mean squared error (MSE) and the Pearson correlation coefficient (PCor) between the values of each correlation matrix of the synthetic data and the corresponding correlation matrix of the test data. For the calculation of PCor, we have excluded the diagonal values of ones in the correlation matrices. We can see that although scDesign2 has a higher MSE than SPsimSeq in panels (a) and (c), it achieves a higher PCor than SPsimSeq in panels (a), (b), and the left column of (c).

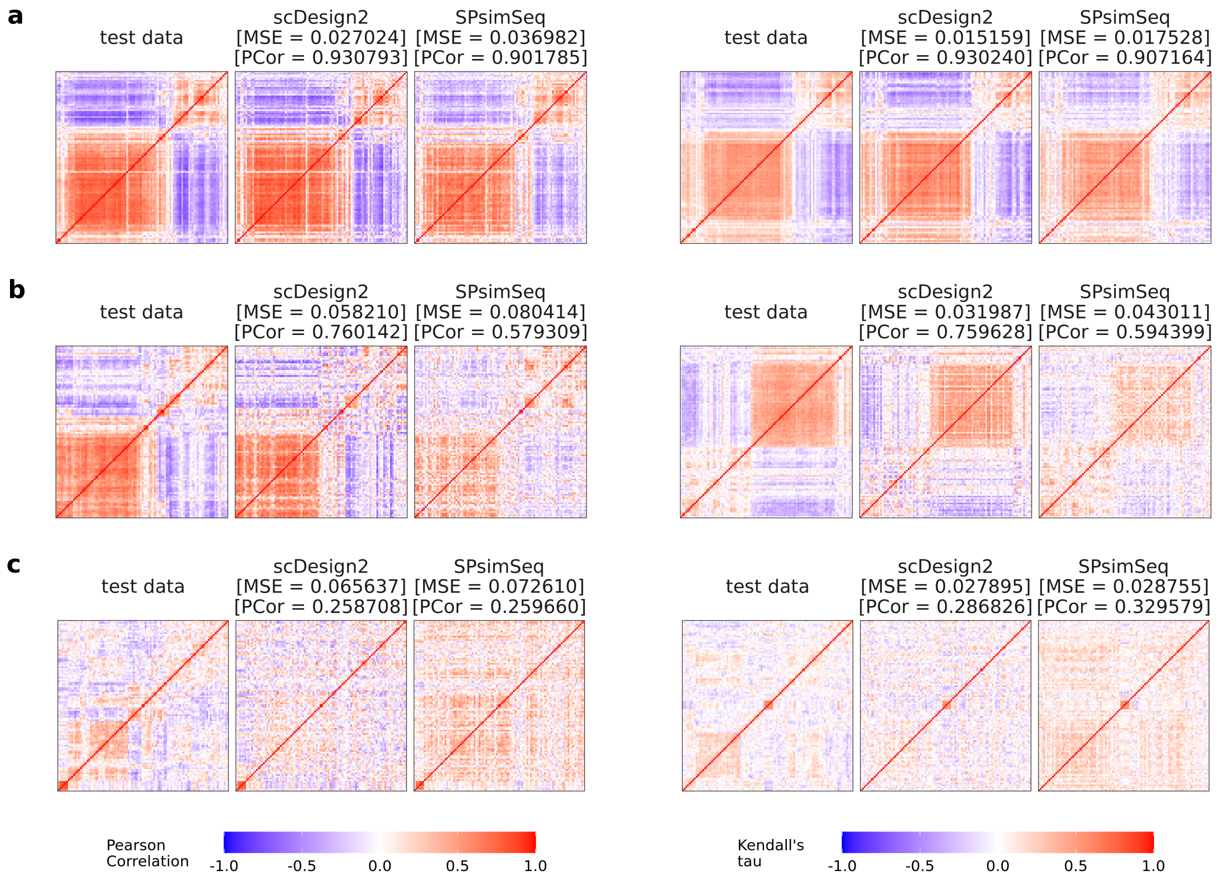

Figure A4: Heatmaps of gene correlation matrices estimated from the test data (real data) and synthetic data generated by scDesign2 and SPsimSeq. The comparison is performed for three cell types from the Smart-Seq2 dataset. The three cell types are (a) dendrocyte (subtype 1), (b) dendrocyte (subtype 2), and (c) monocyte (subtype 2). For each cell type, the Pearson correlation matrices (left column) and Kendall's tau matrices (right column) are shown for the 100 genes with the highest mean expression values in the test data; the rows and columns (i.e., genes) of all the matrices are ordered by the complete-linkage hierarchical clustering of genes (using Pearson correlation as the similarity in the left column and Kendall's tau in the right column) in the test data. We also calculate the mean squared error (MSE) and the Pearson correlation coefficient (PCor) between the values of each correlation matrix of the synthetic data and the corresponding correlation matrix of the test data. For the calculation of PCor, we have excluded the diagonal values of ones in the correlation matrices. We can see that scDesign2 achieves a lower MSE than SPsimSeq in all the comparisons and a higher PCor than SPsimSeq except in panel (c).

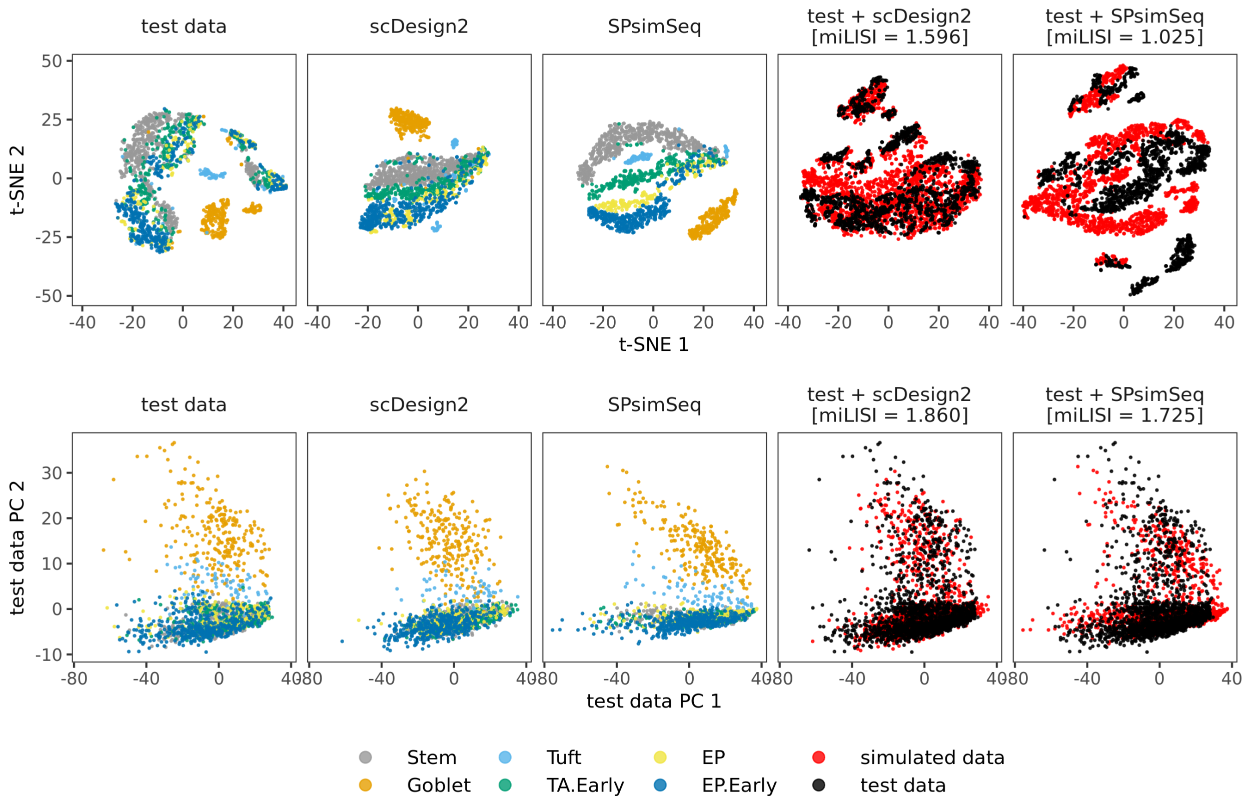

Figure A5: Comparison of 10x Genomics data and synthetic data generated by scDesign2 and SPsimSeq in 2D visualization. t-SNE plots (the first row) and principle component (PC) plots (the second row) of test data, synthetic data generated by the two simulators, and combinations of test data and each synthetic dataset are shown. Note that in the second row, the coordinates are defined by projection to the PC space of the test data. Gene expression counts are transformed as $\log(1 + \text{count})$ before dimensionality reduction. By visually inspecting the patterns in these plots as well as comparing the miLISI values, we find that the synthetic data generated by scDesign2 more resemble the test data.

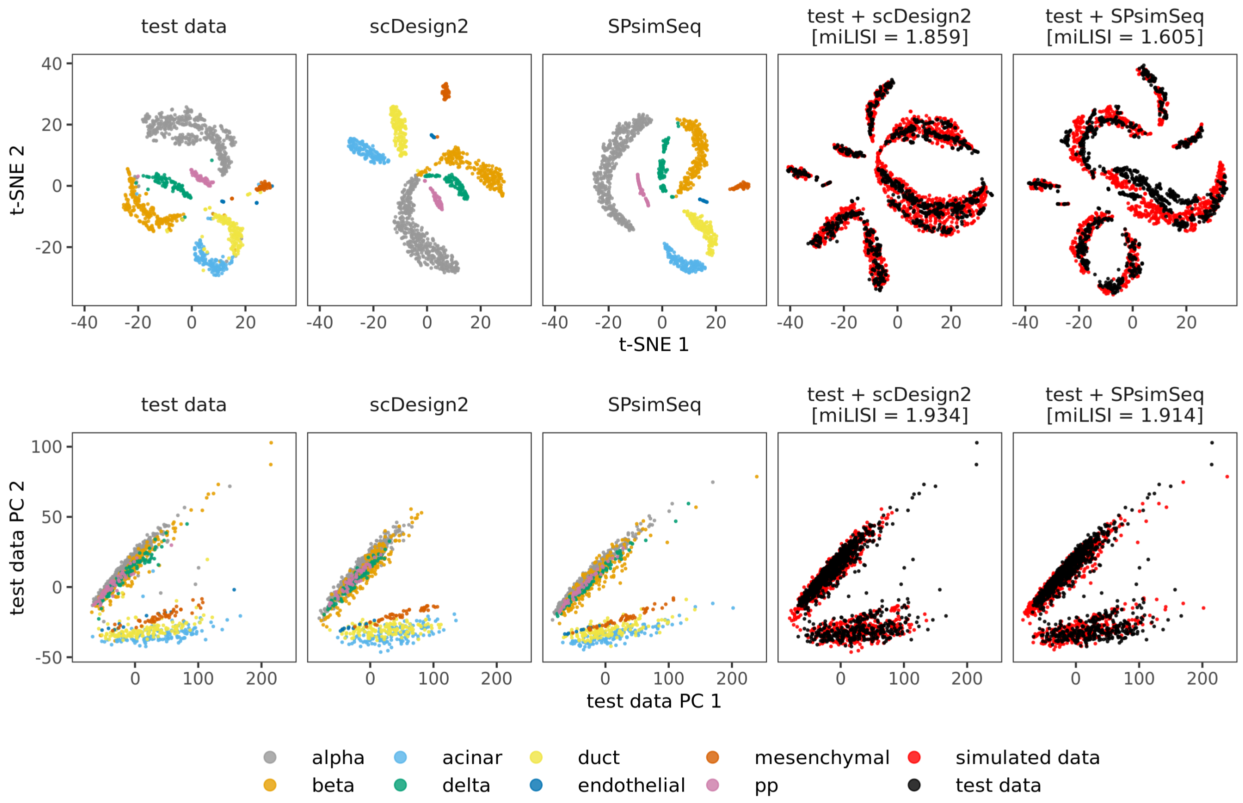

Figure A6: Comparison of CEL-Seq2 data and synthetic data generated by scDesign2 and SPsimSeq in 2D visualization. t-SNE plots (the first row) and principle component (PC) plots (the second row) of test data, synthetic data generated by the two simulators, and combinations of test data and each synthetic dataset are shown. Note that in the second row, the coordinates are defined by projection to the PC space of the test data. Gene expression counts are transformed as $\log(1 + \text{count})$ before dimensionality reduction. By visually inspecting the patterns in these plots as well as comparing the miLISI values, we find that the synthetic data generated by scDesign2 more resemble the test data.

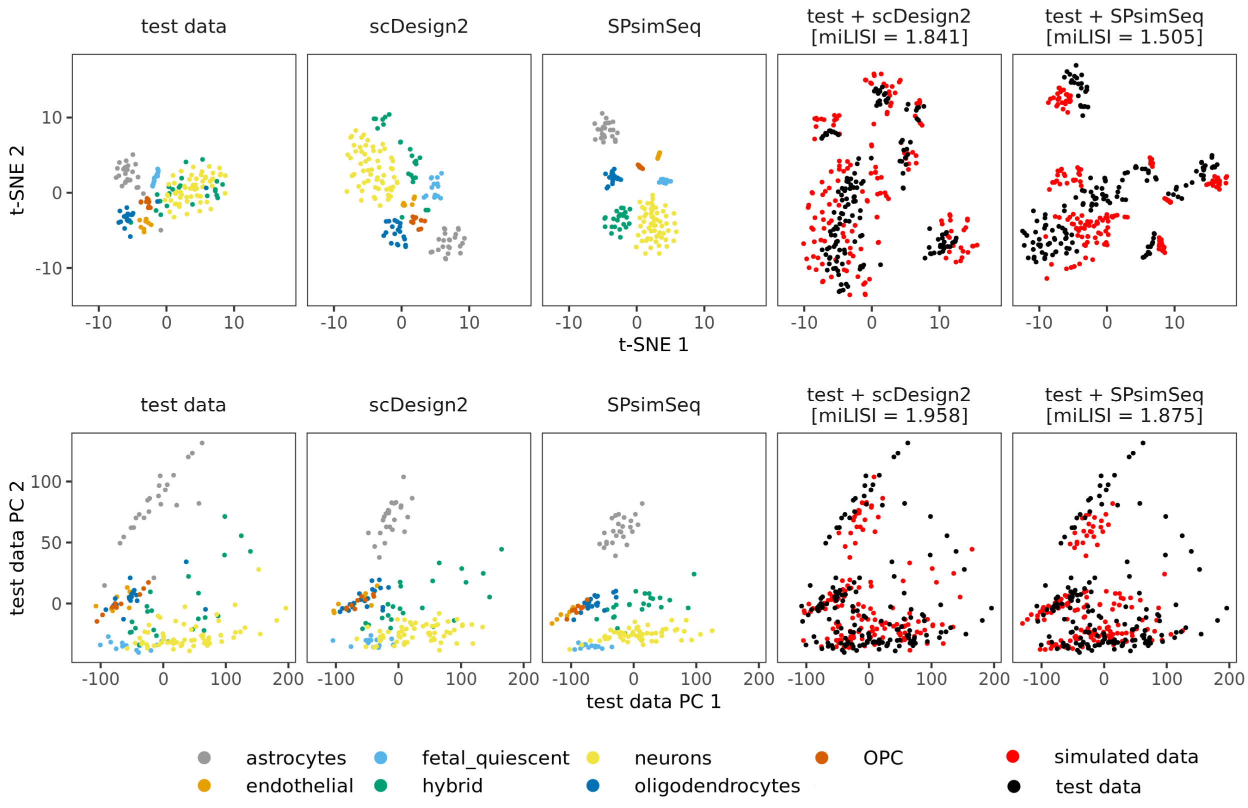

Figure A7: Comparison of Fluidigm C1 data and synthetic data generated by scDesign2 and SPsimSeq in 2D visualization. t-SNE plots (the first row) and principle component (PC) plots (the second row) of test data, synthetic data generated by the two simulators, and combinations of test data and each synthetic dataset are shown. Note that in the second row, the coordinates are defined by projection to the PC space of the test data. Gene expression counts are transformed as $\log(1 + \text{count})$ before dimensionality reduction. By visually inspecting the patterns in these plots as well as comparing the miLISI values, we find that the synthetic data generated by scDesign2 more resemble the test data.

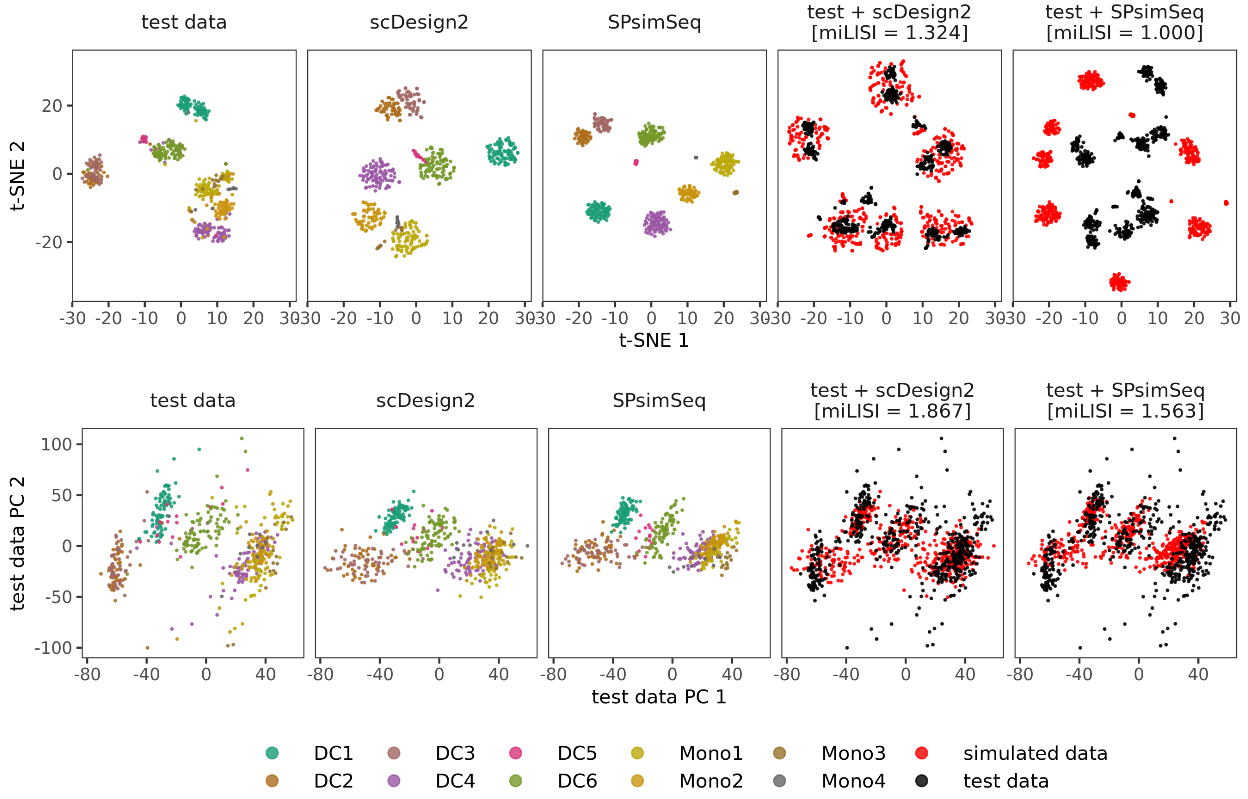

Figure A8: Comparison of Smart-Seq2 data and synthetic data generated by scDesign2 and SPsimSeq in 2D visualization. t-SNE plots (the first row) and principle component (PC) plots (the second row) of test data, synthetic data generated by the two simulators, and combinations of test data and each synthetic dataset are shown. Note that in the second row, the coordinates are defined by projection to the PC space of the test data. Gene expression counts are transformed as $\log(1 + \text{count})$ before dimensionality reduction. By visually inspecting the patterns in these plots as well as comparing the miLISI values, we find that the synthetic data generated by scDesign2 more resemble the test data.

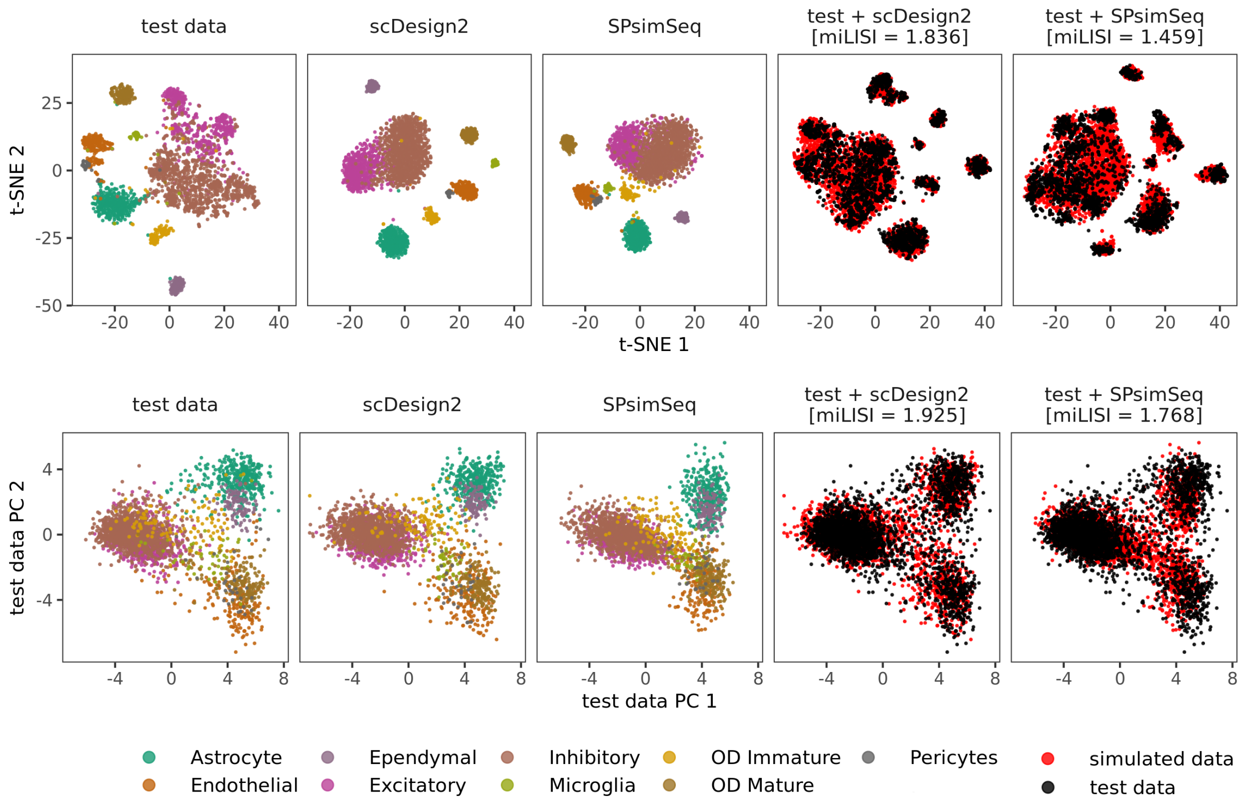

Figure A9: Comparison of MERFISH data and synthetic data generated by scDesign2 and SPsimSeq in 2D visualization. t-SNE plots (the first row) and principle component (PC) plots (the second row) of test data, synthetic data generated by the two simulators, and combinations of test data and each synthetic dataset are shown. Note that in the second row, the coordinates are defined by projection to the PC space of the test data. Gene expression counts are transformed as $\log(1 + \text{count})$ before dimensionality reduction. By visually inspecting the patterns in these plots as well as comparing the miLISI values, we find that the synthetic data generated by scDesign2 more resemble the test data.
