# Supplementary material for: scDesign2: a transparent simulator that generates high-fidelity single-cell gene expression count data with gene correlations captured
Source: Genome Biol. 2021 May 25;22:163. doi: 10.1186/s13059-021-02367-2 (PMC8147071; doi:10.1186/s13059-021-02367-2)
Supplement: Supplementary file 3 — Additional file 3 Review history [file 13059_2021_2367_MOESM3_ESM.docx]

Review History

**First round of review**

**Reviewer 1**

**Were you able to assess all statistics in the manuscript, including the appropriateness of statistical tests used?**

Yes: statistics are appropriate

**Were you able to directly test the methods?**

No

**Comments to author:**

A single-cell RNA-seq (scRNA-seq) simulator that can maximally preserve real data characteristics is very useful for designing scRNA-seq experiments and can be of broad interest. In this manuscript, the authors introduced a new scRNA-seq simulator, scDesign2, that preserves gene, accounts for gene correlation and allows flexibility to specify cell number and sequence depth. The authors compared scDesign2 to a number of existing simulators using data from different scRNA-seq platforms and demonstrated the better performance of scDesign2. They also demonstrated the potential use of this new simulator in optimizing experiment design and comparing computational methods for cell clustering and rare cell type detection. Overall, the methods and results are well-presented, and scDesign2 does show improvements over existing methods. The study and manuscript can be further strengthened by addressing the questions listed below (which can make the results more convincing).

Major:

1. scDesign2 models gene correlation for each cell cluster. This assumes that the cell clustering captures the underlying true cell type structure. However, in real applications obtaining cells' true underlying clustering structure can be difficult. For example, T cells in PBMC have many subtypes. Depending on the data, these subtypes (subclusters) may not always be clearly separable. It is possible that they can be separated by clustering in some datasets but not in others. If the cell clustering used as the input for scDesign2 does not fully capture the true clustering structure, how will the performance of scDesign2 be affected? How sensitive (or robust) is scDesign2 to misspecifications of cells' clustering structure.

2. scDesign2 models gene correlation using sample covariance matrix which has high dimension (p x p where p is the gene number). The cell number in each cluster (particularly for a small cell cluster) may not be enough to support reliable estimation of O(p^2) parameters. I wonder whether modeling the gene correlation, particularly for those small cell clusters, can really help instead of creating overfitting issue.

3. scDesign2 models each cell cluster separately assuming that they are independent. In reality, similar cell clusters (e.g., different subtypes of T cells) may have similar gene correlation structure. Is there a potential way to capture and/or utilize this information? If yes, it will be useful to discuss it. If no, should it be noted as a potential limitation of scDesign2?

4. For Application 1 and Figure 5, it will be useful to have a statistic to quantify the performance of each method. The visual comparison of different methods based on Figure 5 could be misleading due to the potential overlap of different cell types in the two-dimensional embedding (e.g. a purple cell overlapping with an orange cell may be invisible when it is hidden behind the orange cells, yielding a false impression that orange cells form a homogeneous cluster which does not contain any purple cell).

5. For Application 2 and Application 3, when the authors increase the cell number, they fixed the total number of UMIs or reads of the whole dataset (thus each cell will have fewer reads). However, in real applications, one often wants to increase the cell number while holding the sequence depth of each cell unchanged (this will increase the total number of UMIs or reads of the whole dataset). It will be nice if the authors can add such a scenario to their analysis.

6. For all applications (Applications 1-3), what will happen if one does not model gene correlation? It will be useful to compare scDesign2 with scDesign2 without gene correlation. This may help demonstrate why modeling gene correlation is useful. It may also help address my comment #2.

**Reviewer 2**

**Were you able to assess all statistics in the manuscript, including the appropriateness of statistical tests used?**

Yes: The statistics are appropriate.

**Were you able to directly test the methods?**

No

**Comments to author:**

This manuscript proposes scDesign2, an interpretable simulator of scRNA-seq. scDesign2 is a flexible simulator in that it separates models the marginal and joint distribution over the input genes, giving it the flexibility to train on data generated from e.g. one scRNA-seq technology, but simulate according to another (or at least vary sequencing depth, # cells, etc). In the analysis, they benchmarked scDesign2 with existing simulators using scRNA-seq generated under multiple protocols and sequencing technologies to show scDesign2's ability to generate more realistic scRNA-seq data. Their experiments focus on (1) providing guidance for scRNA-seq experimental design, and (2) benchmarking computational tools for the tasks of clustering and rare cell population detection under varying sequence depth and number of cells. The manuscript also suggests other (un-tested) use cases of scDesign2, in benchmarking e.g. DEG methods, as well as performing gene network inference and dataset integration.

Overall, I found the methodological design of scDesign2 interesting, where the covariance structure of genes are preserved in the Gaussian copula, which is novel compared with other simulators. I view this manuscript positively and think it will make a great contribution to the scRNA-seq simulation field. That said, I found the claims of interpretability a bit overstated because the generative process assumed by the model does not necessarily reflect that actual process. Just because the parameters of the model are designed with some phenomena in mind, doesn't necessarily imply that the trained parameters actually reflect those phenomena. Better quantification of some of the results (and less reliance on just visualization) is also useful (see below). I am also not convinced of the utility of scDesign2 for guiding experimental design (see below). In short, it seems like you would need an existing dataset (featuring the same cell types that you want to design an experiment for), in order for you to train scDesign, then vary the 'experiment parameters' to get a better experiment design.

Major comments:

-	I find the interpretability argument of scDesign2 is not compelling. The authors define interpretability as: "its model parameters have biological meanings such as every gene's expression means variance, zero proportions, and every gene pair's expression correlation.". As mentioned above, because the generative process of scDesign2 (as well as any other simulator for that matter) doesn't exactly match the true underlying generative process of scRNA-seq, it's not clear how much the trained parameters reflect the corresponding underlying biological signals. For example, dropout events, sequencing bias, and other technical noise can and will introduce bias, variance into these parameter estimates (or more intuitively, the 'interpretable parameters' will reflect both the phenomena it is designed to capture, as well as e.g. technical artifacts, or other biological phenomena not captured in scDesign2). Without knowing exactly how different scDesign2's generative process is from the real one, it is hard to say how 'interpretable' the parameters are then.

-	In a similar vein, one of the 6 ideal properties (#5) is the interpretability of the simulator. Again, as stated above, it's not clear scDesign2 is actually interpretable. Second, why is this necessary? If the simulator generates 'realistic' scRNA-seq data, why does it matter whether I can look at individual parameters and assign some meaning to it?

-	When scDesign2 is trained on a real dataset, the dataset is first split up into discrete cell types (through use of existing clustering methods), then scDesign2 is trained on each cell type individually. To simulate a new dataset, cell types are sampled roughly proportional to how they are represented in the original training data. These extra steps (training scDesign2 on cell types separately, and explicitly computing and recapitulating cell type proportions in the simulations) would certainly help improve the similarity between simulated and the training data. It's not clear whether the other methods are afforded the same opportunity? Are the other methods trained on each cell type individually, etc? If not (and if it were possible, like for scGAN for example), then the comparison is unfair.

-	Page 6, lines 19-20: the authors note that MSE may not be a great statistic for comparing performance on capturing gene-gene pairwise statistics. Their followup analysis consists of Figs 2c-d (which look like cherrypicked pairs), and Figure 3. Figure 3 is more convincing, but it's not clear why Figure 3 would look good but MSE not, since if MSE is dominated by many values close to 0, that should also be true for Figure 3. Since the correlation matrices are large and the figures are small for Figure 3, I'm left wondering if the MSE (or quantitative measure of accuracy) is the correct one (which suggests not much improvement of scDesign2). The authors should come up with a quantitative metric (other than MSE) to show scDesign2 is better at capturing gene-gene correlations instead of relying on visual confirmation via Figure 3.

-	Figure 4: I think it would be much better to also have a quantitative metric to demonstrate scDesign2 mixes better than the other methods. The authors could just perform a classification experiment (e.g. using cross validation) to quantify how much more 'mixed' scDesign2 is compared to other methods.

-	If I read application #1 correctly, I think the idea of training scDesign2 on one type of data (e.g. scRNA-seq) and simulating another type (e.g. MERFISH) really cool. That said, the MERFISH dataset used by the authors has both scRNA-seq and MERFISH for the same cells --- the authors should train scDesign2 on the scRNA-seq and sample with the same marginals as the MERFISH (subsampled to the same small gene set), then see how indistinguishable the real MERFISH from simulated MERFISH is --- this would provide real dataset validation of the copula.

-	In a similar vein, there have been a number of benchmarking papers published over the past few years where similar cells were sequenced under different platforms. Given one of scDesign's claims is to be able to simulate different platforms for experimental design guidance, I feel a big missing experiment here is using such benchmarking datasets to test to what extent scDesign2 can actually train on one platform and 'simulate' on another.

-	I found Applications #2 and #3 not convincing from the stated goal of guiding experimental design. For example, being able to cluster cells of the same type together, or detect rare cells, depends on a lot of features inherent to a given system (e.g. how close transcriptionally the other cells in the same sample are, what proportion exactly of the population the rare population is). While the goal of scDesign2 in this case makes sense (vary # cells, or depth per cell, to figure out what is needed to distinguish cell types/detect rare cells in my sample of interest), if for example, nobody has sequenced the same cells as I want to (or even under the same conditions), then I have no "real data" to train scDesign2 on (to then simulate). The authors spend quite some effort in Applications #2 and 3 making statements related to the optimal depth and number of cells in their particular experiment, but those numbers are not directly translatable to other experiments that assay other types of cells. The authors need to clearly discuss this limitation up front.

-	I do agree scDesign2 is super useful for benchmarking methods, and determining under what conditions (and likely platforms) one method does better than another. I feel this point is somewhat undersold in the paper, and should be played up.

-	In the methods section, the author proposed an estimator of the covariance matrix R ̂, however the connection between R ̂ and the sample gene-gene covariance structure is not clear, mostly because of the distributional transformation. In particular, for genes with high dropout rate and low expression (for which you would have many ties), the distributional transformation seems like it would introduce a lot of noise. The authors could elaborate by performing simulation experiments to show the connection between covariance matrix R ̂ the sample gene-gene covariance and the underline real gene-gene covariance matrix R (that would be known in simulations).

-	In a similar vein, how does the number of zeros in the gene counts influence R ̂. Although the authors mentioned they only estimate R ̂ for genes that have nonzero entries in more than 20% cells, they didn't provide any guidance on how to select such cutoff.

-	How does sampling u_ij^* influence the estimation of R? Is the result of Figure 3 robust under different sampling of u_ij^*?

Minor comments:

-	Throughout particularly the introduction (e.g. page 2, line 61), the authors use terms like "The simulator can preserve genes so that …". This is a bit confusing, as it's not clear to readers outside the simulation field what it means to "preserve genes". Perhaps "preserve gene features" or "preserve gene structural features" might be easier to read.

-	The authors state the ZINB distribution is a generalization of the others (Poisson, ZIP, NB), but then have a more complex procedure for picking each distribution for each gene during training. Is it worrisome that the MLE procedure can't just fit ZINB distributions to each gene?

-	Page 5, line 40: "matrices have different dimensions" - I understand the genes may not match between simulations and the training data, but this doesn't make the matrices have necessarily different dimensions does it?

-	Page 5, lines 16-50. I personally found it weird to read this large "summary of all figures" before delving into the details of individual figures in the paragraph after that, but that's a matter of personal preference I guess.

-	Page 3, line 6: why do some of the methods need to delete genes? I get that the identity of the genes isn't preserved in some of the simulators, but why do genes need to be deleted?

-	Page 3, line 10: is it more sensible to say "the simulator can capture gene-gene correlations"?

-	Page 3, line 20: may make sense to say "the simulator can generate synthetic data…sequencing depth, while keeping everything else fixed" (e.g. cell identity, etc.).

-	For the 6 summary statistics(mean, variance, cv, genewise-zero proportion, cv and pearson correlation and Kendalls' tau), the author mentioned the computation details of comparison of those summary statistics in Figure2's description. It is worth adding those to the Methods section.

-	For Figure 5, it is worth adding the alignment plots between the simulated dataset and real dataset as Figure 4 did to show the similarity between simulated dataset and real dataset.

-	For visualization purposes, it is worth showing the actual MSE comparison of those summary statistics for 8 simulators rather than simply plotting the distribution.

-	Page 7, last paragraph:

" we still benchmark scDesign2 against ZINB-WaVE and SPARSim, the two simulaors"

Typo: 'simulaors' → 'simulators'

**Authors Response**

**Point-by-point responses to the reviewers’ comments:**

*We thank the two reviewers for their time reviewing our paper and their constructive comments. We have thoroughly revised and enhanced our manuscript based on their comments.*

*In summary, we have made the following eight major changes in our revised manuscript, and these changes are summarized in Figs. 3b, 4, 5, 6, 9, and 12, and Supplementary Figs. S6b-S16b, S17-S32, S35, S38, and S41-S48. In total, we have added 27 new (main and supplement) figures to strengthen our analyses and conclusions.*

*1.	We have included the ROGUE score analysis as a preprocessing step before the model fitting of scDesign2. Users can follow the analysis procedure outlined in the subsection, calibration of cell types with ROGUE scores, on Pages 8-9 to assess the homogeneity of every cell type or cell cluster, before using scDesign2 to fit cell-type-specific models. We have summarized the ROGUE score analysis results in Fig. 5.*

*2.	We have analyzed how the mean squared error (MSE) can be used to evaluate each simulator’s performance on preserving two gene correlation statistics:*

*Pearson correlation and Kendall’s tau.	We have shown in Fig. 3b,*

*Supplementary Figs. S6b-S16b, and Supplementary Figs. S17-S28 that scDesign2 outperforms existing simulators in preserving correlation statistics of the top highly expressed genes. Although when more lowly expressed genes are included for correlation calculation, scDesign2 performs more similar to other simulators, we do not think this phenomenon disproves the advantage of scDesign2. The reason is that the correlations of the top highly expressed genes are key to the resemblance between synthetic data and real data, and that they are more meaningful, both biologically and statistically, than the correlations of the lowly expressed genes. The revised text is in the Results section on Pages 6-7.*

*3.	We have added explanations about the distributional transform used in the estimation of copula correlations. The new text is in the Methods section on Pages 20-21, and a new figure Supplementary Fig. S47 has been added for illustration purposes.*

*4.	We have found an analytical relationship between Kendall’s tau and the Gaussian copula correlation from the literature [1,2]. This relationship offers an interpretation of the copula correlation matrix in the scDesign2 model and is summarized in the Methods section on Page 21.*

*5.	We have designed simulation studies to analyze the effects of n (the sample size, i.e., the number of cells) and p (the number of genes) on the estimation accuracy of the copula correlation matrix. Based on the results, we have added practical suggestions for scDesign2 users and outlined future improvement directions for the scDesign2 method. We have summarized the analysis results in Supplementary Figs. S42 and S43, and we have added text to the Discussion section on Pages 15-16 and the Methods section on Page 22.*

*6.	We have included the median integration LISI (miLISI) as a quantitative metric to assess how well the synthetic data mix with the real data in Figs. 4 and 6, as well as Supplementary Figs. S29-S32. The analysis is summarized in the Results section on Pages 7-8.*

*7.	We have added a scenario for the experimental design application of scDesign2: with the per-cell sequencing depth fixed, the total number of cells sequenced changes. The results are summarized in the Results section on Pages 12 and 14, and Figs. 9 and 12 and Supplementary Figs. S35, S38, and S41.*

*8.	We have demonstrated and explained why the current implementation of scDesign2 cannot be directly used for cross-protocol data simulation. The results are summarized in the Discussion section on Pages 17-18 and in Supplementary Figs. S44-S46.*

Reviewer #1 (Remarks to the Author):

A single-cell RNA-seq (scRNA-seq) simulator that can maximally preserve real data characteristics is very useful for designing scRNA-seq experiments and can be of broad interest. In this manuscript, the authors introduced a new scRNA-seq simulator, scDesign2, that preserves gene, accounts for gene correlation and allows flexibility to specify cell number and sequence depth. The authors compared scDesign2 to a number of existing simulators using data from different scRNA-seq platforms and demonstrated the better performance of scDesign2. They also demonstrated the potential use of this new simulator in optimizing experiment design and comparing computational methods for cell clustering and rare cell type detection. Overall, the methods and results are well-presented, and scDesign2 does show improvements over existing methods. The study and manuscript can be further strengthened by addressing the questions listed below (which can make the results more convincing).

*Response: We thank the reviewer for appreciating the value of our work and providing insightful suggestions.*

Major:

1. scDesign2 models gene correlation for each cell cluster. This assumes that the cell clustering captures the underlying true cell type structure. However, in real applications obtaining cells' true underlying clustering structure can be difficult. For example, T cells in PBMC have many subtypes. Depending on the data, these subtypes (subclusters) may not always be clearly separable. It is possible that they can be separated by clustering in some datasets but not in others. If the cell clustering used as the input for scDesign2 does not fully capture the true clustering structure, how will the performance of scDesign2 be affected? How sensitive (or robust) is scDesign2 to misspecifications of cells' clustering structure.

*Response:*

*We agree with the reviewer’s comment that scDesign2 requires well-defined cell types or clusters in the training data.*

*To refine the cell clustering result so that each cell cluster can be well fit by the scDesign2 model (a necessary condition for generating realistic synthetic data), we have included the ROGUE score analysis. The ROGUE score is proposed in [3] as a metric for assessing the homogeneity of a cell cluster and is associated with the goodness-of-fit by our scDesign2 model.*

*Here is our recommended approach. Before using the ROGUE score, users need to first perform cell clustering with various numbers of clusters (e.g., K=2,3,4...), for example, by controlling the resolution parameter in the Louvain clustering algorithm [4,5], or by directly changing the number of clusters in algorithms such as k-means [6] and SC3 [7]. Second, users need to calculate the ROGUE score for each cell cluster and then take the average ROGUE score across the clusters for each K. The average ROGUE score is expected to increase as K increases because with more clusters, each cluster is expected to be more homogenous. Third, users need to plot the average ROGUE scores against the corresponding K values and choose a reasonable K value (e.g., an “elbow” point, the smallest K value when the average ROGUE score plateaus). Moreover, users can examine the choice of K using t-SNE or UMAP as visual guidance.*

*In summary, we have added the following text (Pages 8-9) and figure (Fig. 5) to the manuscript:*

*“Refinement of scDesign2 training: calibration of cell types by ROGUE scores*

*For a dataset containing multiple cell types, scDesign2 needs to fit a model to each cell type before generating synthetic data. To ensure the quality of its synthetic data, scDesign2 must have one of its count models (ZINB model and its three simplified variants) fit well to each gene’s real expression levels in each cell type; otherwise, the synthetic data may not well mimic real data due to the poorness of model fitting. We observe this issue in the 10x Genomics dataset (Fig. 4a), where some cell types such as transit-amplifying early (TA.Early) cells and goblet cells are composed of discrete sub-clusters in 2D tSNE illustration. As a result, some genes’ expression levels within one of such cell types cannot be fit well by scDesign2’s count models, leading to a discrepancy between synthetic data and real data (synthetic TA.Early and goblet cells do not appear to have cell sub-clusters in 2D tSNE illustration).*

*To address this issue, we calibrate each cell type using the ROGUE score [81], which measures the homogeneity of that cell type, before training scDesign2. Concretely, we first partition the cell type into sub-clusters using the Louvain clustering algorithm [82] in the Seurat R package [41]. Employing varying resolution parameters in the Louvain algorithm, we partition the cell type into a varying number of sub-clusters. Second, we calculate the ROGUE score of every sub-cluster, and then we compute the average ROUGE score across sub-clusters for each number of sub-clusters, ranging from 1 to 6. Third, we examine how the average ROGUE score increases as the number of sub-clusters increases (Fig. 5a), together with 2D t-SNE visualization (Fig. 5b), to determine an appropriate number of sub-clusters, which is usually the “elbow point” where the average ROGUE score saturates.*

*Applying this strategy to refining the six cell types in the 10x Genomics dataset, we observe that, after being trained with the refined cell types, scDesign2 generates more realistic synthetic data (Fig. 5c; the miLISI value increases from 1.596 to 1.779).”*

*Figure 5: Application of ROGUE scores combined with dimensionality reduction plots to refine cell types before training scDesign2. This refinement approach is demonstrated on the 10x Genomics dataset. (a) In each cell type, the relationship between the average ROGUE score across sub-clusters and the number of sub-clusters. Before a ROGUE score is calculated for each sub-cluster, the Louvain clustering algorithm is applied to each cell type with a varying resolution parameter so that a varying number of sub-clusters is obtained. Based on how the average ROGUE score saturates, a number of sub-clusters is selected and marked in red for each cell type. (b) The t-SNE plots of each cell type with the sub-clusters, whose number is marked in (a), labelled with distinct colors. (c) The t-SNE plots of training data (top left), test data (bottom left), synthetic data of scDesign2 trained with the refined sub-clusters (middle left) or the original cell types (middle bottom), and combination of test data with each set of synthetic data. Gene expression counts are transformed as log(1 + count) before dimensionality reduction. We find that, after the cell-type refinement, the simulated data of scDesign2 resemble the real data better, as indicated by the higher miLISI value.*

2. scDesign2 models gene correlation using sample covariance matrix which has high dimension (p x p where p is the gene number). The cell number in each cluster (particularly for a small cell cluster) may not be enough to support reliable estimation of O(p^2) parameters. I wonder whether modeling the gene correlation, particularly for those small cell clusters, can really help instead of creating overfitting issue.

*Response:*

*We thank the reviewer for pointing out this critical issue. Indeed, the estimation of the gene correlation matrix is a high-dimensional problem for each cell type/cluster.*

*To partially avoid this problem, we choose to estimate the copula correlation matrix only for the top highly to moderately expressed genes, so that we can reduce p from ~20,000 to a much smaller number. In the revised manuscript, we have included a simulation study to demonstrate why this approach is reasonable (Supplementary Figs. S42 and S43). In the future, as an improvement direction, we will consider implementing sparse estimation [8] for the copula correlation matrix.*

*We would also like to emphasize that our method scDesign2 does not focus on the inference of the gene correlation matrix; instead, we focus on the simulation of synthetic data. From the Results section, where we compared the scDesign2 synthetic data and the real test data (which are not used for training), we observed that the synthetic data resemble the real data in many aspects. This shows that our current approach does not suffer from overfitting.*

*In summary, we have added the following text (Pages 15-16 and 22) and figures (Supplementary Figs. S42 and S43) to the manuscript:*

*Text on Pages 15-16*

*“Since scRNA-seq data typically contain tens of thousands of genes, the*

*estimation of the copula gene correlation matrix is a high dimensional problem. This problem can be partially avoided by only estimating the copula correlation matrix of thousands of moderately to highly expressed genes. We use a simulation study to demonstrate why this approach is reasonable (Supplementary Figs. S42 and S43), and a more detailed discussion is in the Methods section. To summarize, the simulation results suggest that, to reach an average estimation accuracy of ± 0.3 of true correlation values among the top 1000 highly expressed genes, at least 20 cells are needed. To reach an accuracy level of ± 0.2 for the top 1500 highly expressed genes, at least 50 cells are needed. With 100 cells, an accuracy level of ± 0.1 can be reached for the top 200 highly expressed genes, and a slightly worse accuracy level can be reached for the top 2000 genes.*

*In the implementation of the scDesign2 R package, we control the number of genes for which copula correlations need to be estimated by filtering out the genes whose zero proportions exceed a user-specified cutoff. For all the results in this paper, the cutoff is set as 0.8. In Supplementary Table S1, we summarize the number of cells (n), i.e., the sample size, and the number of genes included for copula correlation estimation (p) in each of the 12 datasets used for benchmarking simulators. Based on Supplementary Figs. S42 and S43, we see that p appears to be too large for the CEL-Seq2, Fluidigm C1, and Smart-Seq2 datasets. This suggests that the results in this paper may be further improved by setting a more stringent cutoff for gene selection.*

*For future methodological improvement, there are other ways to address this high-dimensional estimation problem. For example, we can consider implementing sparse estimation (e.g., [91]) for the copula correlation matrix. Moreover, we can build a hierarchical model to borrow information across cell types/clusters. This will be useful for improving the model fitting for small cell types/clusters that may share similar gene correlation structures.”*

*Text on Pages 22*

*“As a practical note, since the data matrix X typically contains tens of thousands of genes, if the sample size, i.e., the number of cells is not large enough, the estimation of the copula correlation matrix can be problematic [91]. Moreover, many genes are too lowly expressed to be detected in scRNA-seq data, making their correlations uninteresting to estimate. For these two reasons, we argue that the copula correlations should only be estimated for a subset of moderately to highly expressed genes.*

*In Supplementary Figs. S42 and S43, we analyze how n (the sample size, i.e., the number of cells) and p (the number of top expressed genes included) affect the estimation of the copula correlation matrix. We use two example datasets: the stem cell data generated by the 10x Genomics protocol and the dendrocyte (subtype 1) data generated by the Smart-Seq2 protocol. For each dataset, we extract the fitted Gaussian copula model for the top 2000 genes with the highest mean expression levels, and we use this model as the ground truth model to generate 1000 samples with a varying n. Then we estimate the copula correlation matrix of a varying p from each sample. For computational efficiency, we use the plug-in estimation method based on sample tau values: Rˆ_hl = sin (π/2 * τˆ_hl). Finally, we calculate the mean squared error (MSE) between the estimated copula correlations and the true copula correlations. That is, for each n and p, we have 1000 MSE values.*

*In Supplementary Figs. S42 and S43, from panel (a), we can see that MSEs decrease as n increases. From panel (b), we can see that MSEs increase as p increases, i.e., more lowly expressed genes are included. To ease the interpretation of the results, we mark three horizontal lines at MSE = 0.09, 0.04, and 0.01 to represent three levels of estimation quality. On the scale of correlation values, these three levels indicate that on average the estimated values are within ± 0.3, ± 0.2, and ± 0.1 of the true values. The results suggest that to reach the ± 0.3 level of estimation quality, a reasonable choice of n is at least 20, and the top 1000 highly expressed genes can be included. To reach the ± 0.2 level, a reasonable choice of n is at least 50, and the top 1500 highly expressed genes can be included. For n = 100, the ± 0.1 level can be reached for the top 100-200 highly expressed genes, and even the error level for the top 2000 is close to this level. The results confirm that sample size is not a concern for single-cell data because most cell types contain at least a hundred cells that can be measured by current protocols.”*

*Supplementary Figure S42: The effects of n (the sample size, i.e., the number of cells) and p (the number of top highly expressed genes) on the estimation of the copula correlation matrix in the context of 10x Genomics stem cell data. The mean squared error (MSE) between the estimated copula correlations and the true copula correlations are calculated. For each sample size n, 1000 random samples are simulated from a known Gaussian copula model, which is fitted to the 10x Genomics stem cell data, and each sample is then used to estimate the copula correlation matrix. For computational efficiency, we estimate copula correlations using the formula ρ_z= sin (π/2 * τ) by plugging in the sample tau values. (a) The relationship between MSE and n, for each p varying from 100 to 2000. (b) The relationship between MSE and p, for each n varying from 5 to 100. The vertical axes are on the square-root scale.*

*Supplementary Figure S43: The effects of n (the sample size, i.e., the number of cells) and p (the number of top highly expressed genes) on the estimation of the copula correlation matrix in the context of Smart-Seq2 dendrocytes (subtype 1) data. The mean squared error (MSE) between the estimated copula correlations and the true copula correlations are calculated. For each sample size n, 1000 random samples are simulated from a known Gaussian copula model, which is fitted to the Smart-Seq2 dendrocytes (subtype 1) data, and each sample is then used to estimate the copula correlation matrix. For computational efficiency, we estimate copula correlations using the formula ρ_z= sin (π/2 * τ) by plugging in the sample tau values. (a) The relationship between MSE and n, for each p varying from 100 to 2000. (b) The relationship between MSE and p, for each n varying from 5 to 100. The vertical axes are on the square-root scale.*

3.	scDesign2 models each cell cluster separately assuming that they are independent. In reality, similar cell clusters (e.g., different subtypes of T cells) may have similar gene correlation structure. Is there a potential way to capture and/or utilize this information? If yes, it will be useful to discuss it. If no, should it be noted as a potential limitation of scDesign2?

*Response:*

*We thank the reviewer for pointing out this potential improvement direction of our method.*

*It is indeed a limitation of our current method, and we will defer it to future work. We can build a hierarchical model to borrow information across cell types/clusters. This will be useful for improving the model fitting for small cell types/clusters that may share similar gene correlation structures. We have added the following text to the Discussion section (Page 16):*

*“For future methodological improvement, there are other ways to address this high-dimensional estimation problem. For example, we can consider implementing sparse estimation (e.g., [91]) for the copula correlation matrix. Moreover, we can build a hierarchical model to borrow information across cell types/clusters. This will be useful for improving the model fitting for small cell types/clusters that may share similar gene correlation structures.”*

4.	For Application 1 and Figure 5, it will be useful to have a statistic to quantify the performance of each method. The visual comparison of different methods based on Figure 5 could be misleading due to the potential overlap of different cell types in the two-dimensional embedding (e.g. a purple cell overlapping with an orange cell may be invisible when it is hidden behind the orange cells, yielding a false impression that orange cells form a homogeneous cluster which does not contain any purple cell).

*Response:*

*We have remade the original Fig. 5, and now the results are summarized into Fig. 6 and Supplementary Fig. S32 for the two spatial transcriptome technologies MERFISH and pciSeq, respectively. To evaluate the quality of synthetic data in a quantitative way, we have added a quantitative metric for each 2D visualization plot of synthetic and real cells combined. The metric is the median integration local inverse Simpson’s Index (miLISI), whose higher value represents that the synthetic data mix better with the real data in the 2D combination plot. From Fig. 6 and Supplementary Fig. S32, we can see that scDesign2 consistently achieves the highest miLISI. This shows that the scDesign2 synthetic datasets best resemble the corresponding real data of MERFISH and pciSeq.*

*The detailed explanation of miLISI is on Pages 7-8:*

*“To quantify the similarity between synthetic cells and real test cells, we use the median integration local inverse Simpson’s index (miLISI) [80], whose value is between 1 and 2, with a larger value indicating a greater similarity. Specifically, we compute an integration local inverse Simpson’s index (iLISI) to represent the effective number of cell labels (with 1 meaning synthetic or real cells only, and 2 meaning equal numbers of synthetic and real cells) in the local neighborhood of each (synthetic or real) cell; the closer iLISI is to 2, the more equal presence synthetic and real cells have in the local neighborhood. Taking the median of the iLISIs of all cells, we obtain the miLISI, which quantifies the overall mixing of synthetic cells with real cells. Using the R package LISI [80], we calculate the miLISI value for each of the overlaying 2D plots containing real and synthetic cells (Fig. 4 and Supplementary Figs. S29–S31), and we find that scDesign2 consistently leads to the highest miLISI value, with greater advantages in 2D tSNE plots than 2D PCA plots. Since 2D t-SNE projection preserves cell clusters better than 2D PCA does and is more widely used for visualizing single-cell gene expression data, our results suggest that the synthetic data by scDesign2 best capture the cluster structure in real cells. Together, the miLISI values confirm the superb performance and the realistic nature of scDesign2.”*

*The revised analysis for Application 1 can be found on Page 9, Fig. 6, and Supplementary Fig. S32:*

*Text on Page 9*

*“We demonstrate the accuracy of scDesign2 based on two single-cell spatial transcriptome datasets: one dataset of cells in the mouse hypothalamic preoptic region measured by multiplexed error robust fluorescence in situ hybridization (MERFISH) [85] and another dataset of cells in the mouse hippocampal area CA1 measured by probabilistic cell typing by in situ sequencing (pciSeq) [86], a newly developed spatial transcriptome profiling technology. Both datasets contain labeled cell types. Due to the lack of simulators specifically designed for single-cell spatial transcriptome data, we still benchmark scDesign2 against its w/o copula variant, as well as ZINB-WaVE and SPARSim, the two simulators that preserve genes. Note that for all the simulators considered, they only generate gene counts, not spatial coordinates, for synthetic cells. Similar to our previous analysis, for each cell type in each dataset, we randomly split the cells into two halves, with one half (“training data”) to be used for training every simulator and the other half (“test data”) to serve as the benchmark standard to be compared with the synthetic data generated by each simulator. Fig. 6 and Supplementary Fig. S32 demonstrate the 2D visualization of each real dataset, the corresponding synthetic data generated by each simulator, as well as the combination of test data and each synthetic dataset. For both technologies and in both t-SNE and PCA visualization, scDesign2 outperforms SPARSim and ZINB-WaVE by generating synthetic data that most resemble the real data. In particular, scDesign2 consistently achieves the highest miLISI values in the 2D visualization plots of combined data, indicating that the synthetic cells generated by scDesign2 are least distinguishable from real cells. These results confirm the versatility and robustness of scDesign2.”*

*Figure 6: Comparison of MERFISH data and synthetic data generated by scDesign2, its variant without copula, ZINB-WaVE, and SPARSim in 2D visualization. (a) t-SNE plots and (b) principle component (PC) plots of training data, test data, synthetic data generated by each simulator, and combinations of test data and each synthetic dataset. Gene expression counts are transformed as log(1 + count) before dimensionality reduction. miLISI is short for median integration local inverse Simpson's Index, a higher value of which indicates that the simulated data mix better with the test data in the 2D visualization plot. By visually inspecting the patterns in these plots as well as comparing the miLISI values, we find that the synthetic data generated by scDesign2 most resemble the test data.*

*Supplementary Figure 32: Comparison of pciSeq data and synthetic data generated by scDesign2, its variant without copula, ZINB-WaVE, and SPARSim in 2D visualization. (a) t-SNE plots and (b) principle component (PC) plots of training data, test data, synthetic data generated by each simulator, and combinations of test data and each synthetic dataset. Gene expression counts are transformed as log(1 + count) before dimensionality reduction. miLISI is short for median integration local inverse Simpson's Index, a higher value of which indicates that the simulated data mix better with the test data in the 2D visualization plot. By visually inspecting the patterns in these plots as well as comparing the miLISI values, we find that the synthetic data generated by scDesign2 most resemble the test data.*

5. For Application 2 and Application 3, when the authors increase the cell number, they fixed the total number of UMIs or reads of the whole dataset (thus each cell will have fewer reads). However, in real applications, one often wants to increase the cell number while holding the sequence depth of each cell unchanged (this will increase the total number of UMIs or reads of the whole dataset). It will be nice if the authors can add such a scenario to their analysis.

*Response:*

*We thank the reviewer for suggesting this new experimental design scenario. In the revised manuscript, this is referred to as the “fixing the per-cell average sequencing depth” scenario. We have performed analyses for this scenario, and the results are summarized in the following text (Pages 12 and 14), Figs. 9 and 12, and Supplementary Figs. S35, S38, and S41.*

*Text on Page 12*

*“For the third, fixing-average-sequencing-depth scenario, we expect the clustering accuracy to improve as the cell number (and also the total sequencing depth) increases, because more cells will make the identification of cell types easier. Moreover, we expect there to be a saturation effect: the clustering accuracy no longer improves much after the cell number increases to a point, which we regard as the optimal cell number that balances clustering accuracy and budget. The results confirm our expectation. In all four protocols, we observe the expected trend of clustering accuracy for both Seurat and SC3, as well as the saturation effect, which is more obvious for Seurat. In detail, the saturation for 10x Genomics data occurs at 948 cells (based on Seurat), or 3793 cells (based on SC3), while the real dataset has 3,793 cells (Fig. 9); the saturation for CEL-Seq2 data occurs at 1,140 cells, while the real dataset has 2,279 cells (Fig. S35); the saturation for Fluidigm C1 data occurs at 317 cells (based on Seurat), which is the same cell number as the real dataset, while the optimal clustering accuracy occurs at 1,268 cells based on SC3 (Fig. S38); the saturation for Smart-Seq2 data occurs at 4,312 cells, while the real dataset has 1,078 cells (Fig. S41). In conclusion, when the average read (or UMI) count per cell is kept as fixed, for clustering purpose, we recommend keeping the cell number as in the original design for 10x Genomics and using SC3 for clustering; for CEL-Seq2, we recommend decreasing the cell number to 1,140 to save budget and using Seurat for clustering; for Fluidigm C1, if budget allows, we recommend increasing the cell number to 1,268 and using SC3 for clustering; for Smart-Seq2, if budget allows, we recommend increasing the cell number to 4,312 and using either Seurat or SC3 for clustering.”*

*Text on Page 14*

*“For the third, fixing-average-sequencing-depth scenario, we expect the detection accuracy to first increase and then saturate as the cell number increases, similar to our expectation for cell clustering. The detection accuracy of FiRE roughly confirms our expectation, while the detection accuracy of GiniClust2 deviates from this trend (Fig. 12). For FiRE, across thirteen cell numbers ranging from 29 to 121,376 (with the average sequencing depth fixed as 7.53k UMIs per cell, the same as in real data), the F1-score reaches an early local maximum at 474 cells, and then stays relatively stable.*

*A similar trend can be seen for the other three accuracy measures: precision, recall, and AUPRC. For GiniClust2, across nine cell numbers ranging from 29 to 7,586, the F1-score reaches a global maximum at 237 cells, and then it decreases as the cell number further increases. This is mainly due to the increasing proportion of FPs in the discovered rare cells, as indicated by the plunging precision curve. The recall, on the other hand, stays relatively stable after the optimal cell number. The t-SNE visualization supports the observed trends of these accuracy measures. For example, we can see that for GiniClust2, when the cell number reaches 1000, more cells are labelled as FP, as shown in subpanels (4)-(6) of Fig. 12b. In summary, if the goal is to detect tuft cells and the average sequencing depth is fixed as 7.53k UMIs per cell, we recommend using GiniClust2 and decreasing the number of cells to 237.”*

*Figure 9 (Similar figures for other protocols are Supplementary Figs. S35, S38, and S41): scDesign2 guides the choice of cell number in cell clustering, in the case where the average sequencing depth is kept as fixed. scDesign2 generates synthetic 10x Genomics data with eleven cell numbers. Two cell clustering methods---Seurat and SC3---are applied to each synthetic dataset to partition cells into cell clusters. (a) t-SNE visualization of four synthetic datasets, where cells are labelled by Seurat clusters (top), SC3 clusters (middle), and annotated cell types (bottom). (b) Two clustering accuracy measures (AMI and ARI) vs. sequencing depth; left: Seurat; right: SC3. In (b), the results of the four cell numbers in (a) are marked as dots and in the top, and the cell number of the real dataset [76] is marked as vertical dashed lines.*

*Figure 12: scDesign2 guides the choice of cell number in rare cell type detection, in the case where the average sequencing depth is kept as fixed. scDesign2 generates synthetic 10x Genomics data with thirteen cell numbers. Two rare-cell-type detection methods---FiRE and GiniClust2---are applied to each synthetic dataset to detect rare cell types. (a) t-SNE visualization of six synthetic datasets and identification results---true positive (TP), false positive (FP), false negative (FN), and true negative (TN) cells---of FiRE in each dataset. (b) t-SNE visualization of the same six synthetic datasets and identification results of GiniClust2 in each dataset. (c) Four identification accuracy measures by FiRE (precision, recall, F1-score, and AUPRC) vs. cell number. (d) Three identification accuracy measures by GiniClust2 (precision, recall, and F1-score) vs. cell number. In (c) and (d), the results of the six cell numbers in (a) and (b) are marked as dots and in the top, and the cell number of the real dataset [76] is marked as vertical dashed lines. Whenever there is no line for a cell number, FiRE or GiniClust2 does not detect any rare cells or fails.*

6. For all applications (Applications 1-3), what will happen if one does not model gene correlation? It will be useful to compare scDesign2 with scDesign2 without gene correlation. This may help demonstrate why modeling gene correlation is useful. It may also help address my comment #2.

*Response:*

*In our previous manuscript, we referred to scDesign2 without gene correlation as “scDesign2 w/o copula,” and we already included it for benchmarking purposes, for the same rationale as the reviewer suggested. Please see Figs. 2, 3, 4, and 6, and Supplementary Figs. S1-S16, and S29-S32, which show that scDesign2 clearly outperforms its w/o copula variant, confirming the importance of modeling gene correlations. For space consideration, we left out the scDesign2 w/o copular variant from subsequent comparisons.*

Reviewer #2 (Remarks to the Author):

This manuscript proposes scDesign2, an interpretable simulator of scRNA-seq. scDesign2 is a flexible simulator in that it separates models the marginal and joint distribution over the input genes, giving it the flexibility to train on data generated from e.g. one scRNA-seq technology, but simulate according to another (or at least vary sequencing depth, # cells, etc). In the analysis, they benchmarked scDesign2 with existing simulators using scRNA-seq generated under multiple protocols and sequencing technologies to show scDesign2's ability to generate more realistic scRNA-seq data. Their experiments focus on (1) providing guidance for scRNA-seq experimental design, and (2) benchmarking computational tools for the tasks of clustering and rare cell population detection under varying sequence depth and number of cells. The manuscript also suggests other (un-tested) use cases of scDesign2, in benchmarking e.g. DEG methods, as well as performing gene network inference and dataset integration.

Overall, I found the methodological design of scDesign2 interesting, where the covariance structure of genes are preserved in the Gaussian copula, which is novel compared with other simulators. I view this manuscript positively and think it will make a great contribution to the scRNA-seq simulation field. That said, I found the claims of interpretability a bit overstated because the generative process assumed by the model does not necessarily reflect that actual process. Just because the parameters of the model are designed with some phenomena in mind, doesn't necessarily imply that the trained parameters actually reflect those phenomena. Better quantification of some of the results (and less reliance on just visualization) is also useful (see below). I am also not convinced of the utility of scDesign2 for guiding experimental design (see below). In short, it seems like you would need an existing dataset (featuring the same cell types that you want to design an experiment for), in order for you to train scDesign, then vary the 'experiment parameters' to get a better experiment design.

*Response: We want to thank the reviewer for the recognition of our work, as well as the many constructive suggestions.*

Major comments:

1. I find the interpretability argument of scDesign2 is not compelling. The authors define interpretability as: "its model parameters have biological meanings such as every gene's expression means variance, zero proportions, and every gene pair's expression correlation.". As mentioned above, because the generative process of scDesign2 (as well as any other simulator for that matter) doesn't exactly match the true underlying generative process of scRNA-seq, it's not clear how much the trained parameters reflect the corresponding underlying biological signals. For example, dropout events, sequencing bias, and other technical noise can and will introduce bias, variance into these parameter estimates (or more intuitively, the 'interpretable parameters' will reflect both the phenomena it is designed to capture, as well as e.g. technical artifacts, or other biological phenomena not captured in scDesign2). Without knowing exactly how different scDesign2's generative process is from the real one, it is hard to say how 'interpretable' the parameters are then.

*Response:*

*We thank the reviewer for raising the issue about interpretability, and we agree with the reviewer’s point that no simulator trained on scRNA-seq data can retrospectively capture the real biochemical process of scRNA-seq data generation. To clarify, we did not intend to use the term “interpretability” to mean anything about biological mechanisms in our previous version; instead, we wanted to say that our simulator is more transparent and easier to interpret from a modeling perspective than several existing simulators that use deep neural networks. In light of this, we have changed “interpretability” to “transparency” throughout our manuscript, including the title, and clarified our definition of “transparency” (see our response to the next comment below).*

2. In a similar vein, one of the 6 ideal properties (#5) is the interpretability of the simulator. Again, as stated above, it's not clear scDesign2 is actually interpretable. Second, why is this necessary? If the simulator generates 'realistic' scRNA-seq data, why does it matter whether I can look at individual parameters and assign some meaning to it?

*Response:*

*As we replied above, we have changed this property from “interpretability” to “transparency.” We think model transparency is important for computational researchers because without transparency, model diagnostics and improvement would become difficult. With a transparent model, we can easily access how well the model fits to each gene’s marginal distribution and what genes’ correlations are well captured or missed. Hence, whenever the synthetic data do not resemble the real data, we can diagnose the issue and improve the model in a principled way. We deem this a key advantage of using simple probabilistic models over complicated models with too many parameters to interpret. Moreover, our transparent model offers users an opportunity to generate data from their specified parameter values. For example, we received an email inquiry about whether it is possible to generate scRNA-seq data from parameters (gene means) learned from bulk RNA-seq data. While this task cannot be easily achieved with a complicated model, which may have many parameters related to each gene’s mean, it can be done with our model, which only has one parameter for each gene’s mean. The following is the revised text (Page 3) for the “transparency” property.*

*“5. The simulator is transparent so that its model parameters can be easily understood and adjusted. For example, key statistical properties, such as every gene' expression mean, variance, zero proportions, and every gene pair's expression correlation, can be easily accessed from the model. This property is essential for model diagnostics and customized simulation. Specifically, with a transparent model, whenever the synthetic data do not resemble the real data, computational researchers can easily access how well the model fits to each gene’s marginal distribution and what genes’ correlations are well captured or missed. Moreover, a transparent model offers users an opportunity to generate data from their specified parameter values, e.g., gene expression means.”*

3.	When scDesign2 is trained on a real dataset, the dataset is first split up into discrete cell types (through use of existing clustering methods), then scDesign2 is trained on each cell type individually. To simulate a new dataset, cell types are sampled roughly proportional to how they are represented in the original training data. These extra steps (training scDesign2 on cell types separately, and explicitly computing and recapitulating cell type proportions in the simulations) would certainly help improve the similarity between simulated and the training data. It's not clear whether the other methods are afforded the same opportunity? Are the other methods trained on each cell type individually, etc? If not (and if it were possible, like for scGAN for example), then the comparison is unfair.

*Response:*

*We clarify that these steps are also performed for the other simulators. Below is the text quoted from the first paragraph of Page 5.*

*“For each dataset, we randomly split its cells into two halves, with one half (“training data”) to be used for training every simulator on each cell type individually, and the other half (“test data”) to serve as the benchmark standard to be compared with the synthetic data generated by each simulator.”*

4.	Page 6, lines 19-20: the authors note that MSE may not be a great statistic for comparing performance on capturing gene-gene pairwise statistics. Their followup analysis consists of Figs 2c-d (which look like cherrypicked pairs), and Figure 3. Figure 3 is more convincing, but it's not clear why Figure 3 would look good but MSE not, since if MSE is dominated by many values close to 0, that should also be true for Figure 3. Since the correlation matrices are large and the figures are small for Figure 3, I'm left wondering if the MSE (or quantitative measure of accuracy) is the correct one (which suggests not much improvement of scDesign2). The authors should come up with a quantitative metric (other than MSE) to show scDesign2 is better at capturing gene-gene correlations instead of relying on visual confirmation via Figure 3.

*Response:*

*We thank the reviewer for the careful examination and the important question. We have changed the metric from the MSE of all genes’ correlations to the MSE of the 500 most highly expressed genes (in terms of their mean expression levels across cells). We focus on these genes because they explain more than 50% of all the UMIs or reads (Supplementary Figs S17c - S28c). Moreover, as more lowly expressed genes are included, the MSEs of all the simulators decrease and become less distinguishable (because lowly expressed gene pairs have correlations close to zero in test data and all synthetic data), making the comparison less meaningful.*

*The c-d panels of Fig. 3 (the original Fig. 2) are two examples where scDesign2 can preserve strong negative gene correlations while the other two simulators cannot. This advantage can be seen from the last two panels of the violin plots in Fig. 3a; Figs. 3c-d are just two non-cherry-picked examples showing this phenomenon.*

*The revised text about this MSE metric can be found on Pages 6-7. Fig. 3b and Supplementary Figs. S6b-S16b (which correspond to the original Supplementary Figs. S1b-S11b) have been revised accordingly. Supplementary Figs. S17-S28 have been added to demonstrate the relationship between MSE and the number of highly expressed genes included for correlation estimation.*

*Text on Pages 6-7*

*“Out of 24 comparisons of gene-pair-wise statistics (2 correlation statistics times 12 cell-type–protocol combinations) based on the 500 most highly expressed genes (in terms of their mean expression levels across cells) in each cell-type–protocol combination, scDesign2 achieves the best MSEs in 15 comparisons (Fig. 3b and Supplementary Figs. S6b–S16b). We highlight the highly expressed genes because their Pearson correlations and Kendall’s tau values are more biologically meaningful; in all cell-type–protocol combinations, the top 500 highly expressed genes, ranked by either mean expression levels or non-zero proportions across cells, explain at least 50% of reads or UMIs (Supplementary Figs. S17c–S28c), confirming that these genes play dominant roles in transcriptional programs in cells. In addition, we include the comparison results based on more genes in Supplementary Figs. S17d&e–S28d&e, which show that, as more lowly expressed genes are included, the MSEs of all these simulators decrease and become less distinguishable (because lowly expressed gene pairs have correlations close to zero in test data and all synthetic data), making the comparison less meaningful.”*

*Figure 3 (Similar figures for other protocols are Supplementary Figs. S6-S16; only panel b has been revised): Benchmarking scDesign2 against its variant without copula and eight existing scRNA-seq simulators for generating goblet cells measured by 10x Genomics. ... (b) Ranking (with 1 being the best-performing method) of scDesign2, ZINB-WaVE, SPARSim, and scGAN, the only four methods that preserve genes, in terms of the mean-squared error (MSE) of each of six summary statistics (four gene-wise and two gene-pair-wise) between the statistic values in the real data and the synthetic data generated by each simulator. Note that the color scale shows the normalized MSE: for each statistic (column in the table), the normalized MSEs are the MSEs divided by the largest MSE of that statistic. scDesign is ranked the top for three out of the six statistics. For the two gene-pair-wise statistics, we focus on the top 500 highly expressed genes, because as analyzed in the text, they are more meaningful, both biologically and statistically, than the correlations of the lowly expressed genes....*

*Supplementary Figure S17 (Similar figures for other protocols are Supplementary Figs. S18-S28): Relationships of the mean squared error (MSE) vs. the dimension (i.e., number of genes) of the (Pearson or Kendall's tau) gene correlation matrices, which are estimated from the synthetic data generated by four simulators trained on the 10x Genomics goblet cell data. The genes are ordered in two ways, by their mean expression from high to low (left) or by their zero proportion from low to high (right). The plots are shown for the top 100, 200, 500, 1000, 1500, and 2000 genes ordered in either way. (a) The zero proportion of each top gene. (b) The mean expression of each top gene. (c) The relationship of the cumulative proportion of the top genes' UMIs among all UMIs vs. the number of top genes. (d)-(e) The MSE is defined as the average per-entry squared difference between the correlation matrices estimated from each synthetic dataset and the test data. Each plot shows the relationship of the MSE of (d) the Pearson correlation matrix or (e) the Kendall's tau matrix for the top genes estimated from each simulator's synthetic data vs. the number of top genes.*

5.	Figure 4: I think it would be much better to also have a quantitative metric to demonstrate scDesign2 mixes better than the other methods. The authors could just perform a classification experiment (e.g. using cross validation) to quantify how much more 'mixed' scDesign2 is compared to other methods.

*Response:*

*We thank the reviewer for this suggestion. We have added a metric called the median integration local inverse Simpson's index (miLISI) [9] to quantify the mixing of the synthetic data and the real data. The miLISI values have been added to Figs. 4-6 and Supplementary Figs. S29-S32, and S44.*

*We have added to Pages 7-8 the description about the miLISI metric, and how it shows that scDesign2’s synthetic data mix better with real data than the other simulators’ synthetic do. The quoted text can be found on Page 11 of this response letter.*

6.	If I read application #1 correctly, I think the idea of training scDesign2 on one type of data (e.g. scRNA-seq) and simulating another type (e.g. MERFISH) really cool. That said, the MERFISH dataset used by the authors has both scRNA-seq and MERFISH for the same cells --- the authors should train scDesign2 on the scRNA-seq and sample with the same marginals as the MERFISH (subsampled to the same small gene set), then see how indistinguishable the real MERFISH from simulated MERFISH is --- this would provide real dataset validation of the copula.

*Response:*

*We apologize for the confusion. For application 1, we did not perform cross-protocol simulation. For the MERFISH data simulation, we trained scDesign2 on MERFISH data, not scRNA-seq data. Moreover, we only used the count data for model training and did not use the spatial coordinates.*

*The revised analysis for application 1 can be found on Page 9 and Fig. 6 and Supplementary Fig. S32 in the revised manuscript. The quoted text and the corresponding figures can be found on Pages 11-13 of this response letter.*

7.	In a similar vein, there have been a number of benchmarking papers published over the past few years where similar cells were sequenced under different platforms. Given one of scDesign's claims is to be able to simulate different platforms for experimental design guidance, I feel a big missing experiment here is using such benchmarking datasets to test to what extent scDesign2 can actually train on one platform and 'simulate' on another.

*Response:*

*Although scDesign2 can model and simulate data from different scRNA-seq protocols and other single-cell expression count-based technologies, the current scDesign2 implementation is not yet applicable to cross-protocol data generation (i.e., training scDesign2 on real data of one protocol and generating synthetic data for another protocol) because of complicated differences in data characteristics among different protocols.*

*To demonstrate this issue, we have performed a cross-protocol data generation experiment, using a multi-protocol dataset of peripheral blood mononuclear cells (PBMCs) generated for benchmarking purposes [10]. To further illustrate the different data characteristics of different protocols, we have compared individual genes' mean expression levels measured by three protocols for the same batch of PBMCs.*

*The results of the above analysis can be found in the following text (Pages 17-18) and Supplementary Figs. S44-S46:*

*“Regarding the use of scDesign2 to guide the design of scRNA-seq experiments, although scDesign2 can model and simulate data from different scRNA-seq protocols and other single-cell expression count-based technologies, the current scDesign2 implementation is not yet applicable to cross-protocol data generation (i.e., training scDesign2 on real data of one protocol and generating synthetic data for another protocol) because of complicated differences in data characteristics among protocols. To demonstrate this issue, we use a multi-protocol dataset of peripheral blood mononuclear cells (PBMCs) generated for benchmarking purposes [19]. We select data of five cell types measured by three protocols, 10x Genomics, Drop-Seq, and Smart-Seq2, and we train scDesign2 on the 10x Genomics data. Then we adjust the fitted scDesign2 model for the Drop-Seq and Smart-Seq2 protocols by rescaling the mean parameters in the fitted model to account for the total sequencing depth and cell number, which are protocol-specific (see Methods for details). After the adjustment, we use the model for each protocol to generate synthetic data. Supplementary Fig. S44 illustrates the comparison of real data and synthetic data for each protocol. From the comparison, we observe that the synthetic cells do not mix well with the real cells for the two cross-protocol scenarios; only for 10x Genomics, the same-protocol scenario, do the synthetic cells mix well with the real cells.*

*To further illustrate the different data characteristics of different protocols, we compare individual genes’ mean expression levels in the aforementioned three protocols. We refer to Drop-Seq and Smart-Seq2 as the target protocols, and 10x Genomics as the reference protocol. First, we randomly partition the two target-protocol datasets and the reference-protocol dataset into two halves each; we repeat the partitions for 100 times and collect 100 sets of partial datasets, with each set containing two target-protocol partial datasets (one Drop-Seq and one Smart-Seq2) and two reference-protocol partial datasets (split from the 10x-Genomics dataset)—one of the latter is randomly picked and referred to as the “reference data.” Second, For every gene in each cell type, we take each set of partial datasets and compute two cross-protocol ratios, defined as the gene’s mean expression levels in the target-protocol partial datasets divided by its mean expression level in the reference data, and a within-protocol ratio, defined as the ratio of the gene’s mean expression level in the other reference-protocol partial dataset divided by that in the reference data; together, with the 100 sets of partial dataset, every gene in each cell type has 100 ratios for each of the two cross-protocol comparisons and 100 ratios for the within-protocol comparison. We apply this procedure to the top 50 and 2000 highly expressed genes in five cell types. Supplementary Figs. S45 and S46 show that, with the within-protocol ratios as a baseline control for each cell type and each target protocol, the cross-protocol ratios exhibit a strongly gene-specific pattern; moreover, there is no monotone relationship between the cross-protocol ratios and the mean expression levels of genes. This result confirms that there does not exist a single scaling factor to convert all genes’ expression levels from one protocol to another. However, an interesting phenomenon is that, for each target protocol, the cross-protocol ratios have similar patterns across cell types. This phenomenon sheds light on a future research direction of cross-protocol simulation for the cell types that exist in only one protocol, if the two protocols have shared cell types. In this scenario, we may train a model for each cell type in each protocol, learn a gene-specific but cell-type-invariant scaling factor from the shared cell types, and simulate data for the cell types missing in one protocol.”*

*Supplementary Figure S44: 2D t-SNE visualization of the results of a cross-platform simulation experiment. We use a multi-protocol dataset of peripheral blood mononuclear cells (PBMCs) generated for benchmarking purposes [19]. We select data of five cell types measured by three protocols, 10x Genomics, Drop-Seq, and Smart-Seq2, and we train scDesign2 on the 10x Genomics data. Then we adjust the fitted scDesign2 model for the Drop-Seq and Smart-Seq2 protocols by rescaling the mean parameters in the fitted model (see Methods for details). After the adjustment, we use the model for each protocol to generate synthetic data. The 2D t-SNE visualization plot is generated for the real data, the synthetic data, and the combination of the real data and the synthetic data, for each of the three protocols. From the combination plot, we can see that the synthetic cells do not mix well with the real cells for the two cross-protocol scenarios; only for 10x Genomics, the same-protocol scenario, the synthetic cells mix well with the real cells. As a quantitative comparison, the median integration local inverse Simpson's Index (miLISI) for the three combination plots are 1.756, 1.000, and 1.000, from left to right, with the largest value indicating the best mixing for the same-protocol scenario, confirming our conclusion.*

*Supplementary Figure S45: The cross-protocol and within-protocol ratios of genes' mean expression levels between a target protocol (Drop-Seq or Smart-Seq2) and the reference protocol (10x Genomics) in five cell types. The results are shown for the top 50 highly expressed genes across the five cell types in the reference protocol, ordered by mean expression from high to low. In each cell type (row) and each target protocol (column), a gene has 100 cross-protocol ratios and 100 within-protocol ratios as a result of random partitioning. To illustrate the trends of the ratios, smoothed curves are added by the R function geom_smooth(). The calculation detail is as follows. For each cell type, suppose the target protocol has m cells, and the reference protocol has n cells. In each random partition, we first randomly select min(m,n)/2 cells from the target protocol and the reference protocol each, and compute a gene's two means respectively. Second, for the remaining cells in the reference protocol, we randomly select n/2 cells and compute the same gene's mean as a reference. Third, we calculate the cross-protocol ratio and the within-protocol ratio by dividing the first two means by the reference mean. We repeat the above three steps for 100 times for every gene in each cell type and each target protocol. From the plot, we can see that, unlike the within-protocol ratios, which center around the constant value of 1, the cross-protocol ratios fluctuate between genes, and do not center around a constant value. This shows that there does not exist a single scaling factor to convert all genes' expression levels from one protocol to another.*

*Supplementary Figure S46: The cross-protocol and within-protocol ratios of genes' mean expression levels between a target protocol (Drop-Seq or Smart-Seq2) and the reference protocol (10x Genomics) in five cell types. The results are computed for the top 2000 highly expressed genes in each cell type in the reference protocol, ordered by mean expression from high to low. In each cell type (row) and each target protocol (column), a gene has 100 cross-protocol ratios and 100 within-protocol ratios as a result of random partitioning. For clarity of illustration, only the trends of the ratios are shown, which are smoothed curves, added by the R function geom_smooth(). The calculation detail is as follows. For each cell type, suppose the target protocol has m cells, and the reference protocol has n cells. In each random partition, we first randomly select min(m,n)/2 cells from the target protocol and the reference protocol each, and compute a gene's two means respectively. Second, for the remaining cells in the reference protocol, we randomly select n/2 cells and compute the same gene's mean as a reference. Third, we calculate the cross-protocol ratio and the within-protocol ratio by dividing the first two means by the reference mean. We repeat the above three steps for 100 times for every gene in each cell type and each target protocol. From the plot, we can see that, unlike the within-protocol ratios, which center around the constant value of 1, the cross-protocol ratios fluctuate between genes, and do not center around a constant value. This shows that there does not exist a single scaling factor to convert all genes' expression levels from one protocol to another.*

8.	I found Applications #2 and #3 not convincing from the stated goal of guiding experimental design. For example, being able to cluster cells of the same type together, or detect rare cells, depends on a lot of features inherent to a given system (e.g. how close transcriptionally the other cells in the same sample are, what proportion exactly of the population the rare population is). While the goal of scDesign2 in this case makes sense (vary # cells, or depth per cell, to figure out what is needed to distinguish cell types/detect rare cells in my sample of interest), if for example, nobody has sequenced the same cells as I want to (or even under the same conditions), then I have no "real data" to train scDesign2 on (to then simulate). The authors spend quite some effort in Applications #2 and 3 making statements related to the optimal depth and number of cells in their particular experiment, but those numbers are not directly translatable to other experiments that assay other types of cells. The authors need to clearly discuss this limitation up front.

*Response:*

*We thank the reviewer for pointing out this limitation of applications 2 and 3. We have added the following text (Page 18) to the Discussion section to provide a more detailed explanation about what real world situations can these analyses be directly applied.*

*“In applications 2 and 3, we have demonstrated how to use scDesign2 to guide experimental design and benchmark computational methods for the tasks of cell clustering and rare cell type detection. Note that in these analyses, the optimized sequencing depths and cell numbers are only applicable to the same experimental protocols and biological samples. Yet, this limitation does not disqualify scDesign2 as a useful tool to guide experimental design. For example, researchers usually perform a coarse-grained, low-budget experiment to obtain a preliminary dataset, and then they may use scDesign2 to guide the optimal design of the later, more refined experiment. As another example, if scRNA-seq data need to be collected from many individuals, researchers usually first perform a pilot study on a small number of individuals. Then they may train scDesign2 using the pilot data to guide the design of the subsequent, large-scale experiments. Moreover, in addition to guiding the experimental design, scDesign2 is useful as a general benchmarking tool for various experimental protocols and computational methods, as shown in applications 2 and 3. For example, the analyses we performed in applications 2 and 3 are easily generalizable to other computational methods for a more comprehensive benchmarking.”*

9.	I do agree scDesign2 is super useful for benchmarking methods, and determining under what conditions (and likely platforms) one method does better than another. I feel this point is somewhat undersold in the paper, and should be played up.

*Response:*

*We thank the reviewer for recognizing this advantage of scDesign2. Although we do not have a separate subsection that entirely focuses on the benchmarking of computational methods, we have emphasized benchmarking as an important application of scDesign2 in the Introduction section, and we have benchmarked four popular computational methods in applications 2 and 3. The analyses we performed in these two applications are easily generalizable to other computational methods for a more comprehensive benchmarking. We would also like to note that the main goal of our paper is to propose a new simulator, not to perform a benchmarking study.*

*We have included this point in the text on Page 18 in the Discussion section. Please refer to the previous comment.*

10. In the methods section, the author proposed an estimator of the covariance matrix R ,' however the connection between R 'and the sample gene-gene covariance structure is not clear, mostly because of the distributional transformation. In particular, for genes with high dropout rate and low expression (for which you would have many ties), the distributional transformation seems like it would introduce a lot of noise. The authors could elaborate by performing simulation experiments to show the connection between covariance matrix R 'the sample gene-gene covariance and the underline real gene-gene covariance matrix R (that would be known in simulations).

*Response:*

*The goal of the distributional transform is to convert the original discrete random variable into a continuous random variable that has a continuous cumulative distribution function (CDF) with a slight domain change (i.e.,if the original random variable has positive probability masses in the interval [x, y], then the continuous random variable has positive densities in the interval [x, y+1)), so that the copula function can be uniquely identified.*

*Although it introduces additional randomness into the estimation of the copula correlation matrix, it has very little effect on the synthetic data. This can be seen from Supplementary Fig. S48, where the result of Figure 2 (Figure 3 in the original manuscript) does not change much under a different sampling of v_ij^*.*

*We have included the following text (Page 21) and Supplementary Fig. S48 to explain this:*

*Supplementary Figure S48: Heatmaps of gene correlation matrices estimated from synthetic data generated by scDesign2, with R^ estimated under two different random samples of v_ij^*. (a)-(b) Pearson correlation matrices; (c)-(d) Kendall's tau matrices. In (a) and (c), the scDesign2 model is fitted with goblet cells measured by 10x Genomics [76]; In (b) and (d), the scDesign2 model is fitted with cells of dendrocytes subtype 1 (DC1) measured by Smart-Seq2 [79]. For each cell type, the Pearson correlation matrices and Kendall's tau matrices are shown for the 100 genes with the highest mean expression values in the test data; the rows and columns (i.e., genes) of all the matrices are ordered by the complete-linkage hierarchical clustering of genes (using Pearson correlation as the similarity in (a)-(b) and Kendall's tau in (c)-(d)) in the test data. We find that the effect of the sampling of v_ij^* on the estimated gene correlation matrices of the synthetic data is negligible, since the two matrices in each panel are very similar.*

*Moreover, for genes with high dropout rates and low expression, they are excluded from the estimation of the copula correlation matrix, due to the need to address the high-dimensional estimation problem. Details of this strategy can be found on Pages 6-9 of this response letter.*

*From existing literature, we have found an analytical expression that links the Gaussian copula correlations to Kendall's tau values in the original unit of gene expression. Due to the property of copula, such analytical relationships only exist for ranked correlations, not the Pearson correlation, yet they provide a way to interpret the fitted Gaussian copula correlation values.*

*A more detailed discussion of the distributional transform and this analytical relationship has been added to the text (Pages 20-21) and Supplementary Fig. S47.*

*Text on Pages 20-21*

*distributional transform:*

*analytical relationship:*

*Supplementary Figure S47: A toy example showing the effect of the distributional transform. For a discrete random variable X, the distributional transform maps its probability mass at each value x, which has a non-zero probability mass, uniformly to the interval [x, x+1), thus converting X to a continuous random variable. The top left and right panels show the probability mass function (PMF) before the transform and the probability density function (PDF) after the transform; the bottom left and right panels show the cumulative distribution functions (CDFs) before and after the transform.*

11.	In a similar vein, how does the number of zeros in the gene counts influence R ̂. Although the authors mentioned they only estimate R ̂ for genes that have nonzero entries in more than 20% cells, they didn't provide any guidance on how to select such cutoff.

*Response:*

*Similar to the response to comment 10, for genes with high proportion of zeros, the distributional transform randomly distributes the fitted probability of zero into the interval [0, 1) independent of other genes. Due to the need to address the high-dimensional estimation problem, these genes are excluded from the estimation of the copula correlation matrix.*

*In our revised manuscript, after a more detailed analysis of the effects of sample size (n) and dimensionality (p) on the estimation of the copula correlation matrix, we have proposed a new guideline for choosing what genes to include (and exclude) when estimating the copula correlation matrix. Our analysis is summarized in the text on Pages 15-16 and 22 and Supplementary Figs. S42 and S43. The quoted text and figures can be found on Pages 6-9 of this response letter.*

12.	How does sampling u_ij^* influence the estimation of R? Is the result of Figure 3 robust under different sampling of u_ij^*?

*Response:*

*The sampling of v_ij^* introduces additional randomness into the estimation of R. However, this additional randomness has a negligible effect on the synthetic data. This can be seen from Supplementary Fig. S48, where the result of Figure 2 (Figure 3 in the original manuscript) does not change much under a different sampling of v_ij^*.*

*Please refer to Page 32 of this response letter for the revised text (Page 21) and Supplementary Fig. S48.*

Minor comments:

1. Throughout particularly the introduction (e.g. page 2, line 61), the authors use terms like "The simulator can preserve genes so that ...". This is a bit confusing, as it's not clear to readers outside the simulation field what it means to "preserve genes". Perhaps "preserve gene features" or "preserve gene structural features" might be easier to read.

*Response:*

*By preserving genes, we mean that every gene in the simulated data uniquely corresponds to a gene in the real data. In other words, there exists a one-to-one map between the genes in the simulated data and the genes in the real data. Some simulators do not have this property.*

2.	The authors state the ZINB distribution is a generalization of the others (Poisson, ZIP, NB), but then have a more complex procedure for picking each distribution for each gene during training. Is it worrisome that the MLE procedure can't just fit ZINB distributions to each gene?

*Response:*

*The model selection provides useful interpretations. If ZINB or ZIP is selected instead of NB or Poisson, it means that the gene expression distribution is zero-inflated. If ZIP or Poisson is selected instead of ZINB or NB, it means that there is no overdispersion in the gene expression distribution.*

*One application of this model selection procedure is to reveal the technical differences between protocols. We have demonstrated this application with an example in a Perspective paper we wrote about zeros in scRNA-seq data [11]. In this example, we use a multi-protocol dataset of peripheral blood mononuclear cells (PBMCs) generated for benchmarking purposes [10]. We selected data of five cell types measured by three protocols, 10x Genomics, Drop-Seq, and Smart-Seq2 and applied the model fitting and selection procedure for each gene. The results are summarized in Fig. 5d, which shows that non-zero-inflated distributions (Poisson and NB) are chosen for almost all genes in the 10x Genomics and Drop-seq datasets, while zero-inflated distributions (ZIP and ZINB) are chosen for about half of the genes in the Smart-seq2 dataset. This shows that, in the same cell type, the same gene's expression distribution may be described by different statistical models for data generated by different protocols.*

3.	Page 5, line 40: "matrices have different dimensions" - I understand the genes may not match between simulations and the training data, but this doesn't make the matrices have necessarily different dimensions does it?

*Response:*

*We thank the reviewer for pointing out this confusion. We have revised the text (Page 6)*

*to:*

*“the genes of the synthetic data matrix cannot be matched one-to-one to the genes of the training data matrix”*

4.	Page 5, lines 16-50. I personally found it weird to read this large "summary of all figures" before delving into the details of individual figures in the paragraph after that, but that's a matter of personal preference I guess.

*Response:*

*We hope to provide an overview for the readers before delving into too many details. Since we have compared simulators from many aspects, which may be overwhelming, we decide to first summarize the tasks we have performed, and next delve into the details of the analysis and conclusions of each task.*

5.	Page 3, line 6: why do some of the methods need to delete genes? I get that the identity of the genes isn't preserved in some of the simulators, but why do genes need to be deleted?

*Response:*

*This is due to the design of those specific simulators. One common reason is that some genes have too many zeros, so the simulator developers deemed those genes uninteresting to simulate.*

6.	Page 3, line 10: is it more sensible to say "the simulator can capture gene-gene correlations"?

*Response:*

*We thank the reviewer for this comment. However, since we use the term “gene correlations” throughout the manuscript, including the title, and we find it not confusing, we prefer to keep it as is.*

7.	Page 3, line 20: may make sense to say "the simulator can generate synthetic data...sequencing depth, while keeping everything else fixed" (e.g. cell identity, etc.).

*Response:*

*We thank the reviewer for pointing out this inaccuracy. We have added “under the same biological condition of training data” for better clarification. The following is the revised sentence (still on Page 3):*

*The simulator can generate synthetic data with both varying cell number and sequencing depth, under the same biological condition of training data.*

8.	For the 6 summary statistics(mean, variance, cv, genewise-zero proportion, cv and pearson correlation and Kendalls' tau), the author mentioned the computation details of comparison of those summary statistics in Figure2's description. It is worth adding those to the Methods section.

*Response:*

*We have already outlined the key steps of how these summary statistics are calculated in our main text. Only two more computation details are added in the caption, which are (1) in (b), the MSEs are normalized by the largest MSE so that it’s easier to compare the colors across different summary statistics, and (2) in (e), the smoothed curves are plotted by the R function geom_smooth(). Therefore, we do not feel it necessary to add a separate subsection to the Methods section. We feel that, by putting these calculation details directly in the caption, it would be more convenient for the readers to understand this figure.*

9.	For Figure 5, it is worth adding the alignment plots between the simulated dataset and real dataset as Figure 4 did to show the similarity between simulated dataset and real dataset.

*Response:*

*Thanks to the reviewer’s comment, we have added the alignment plots. Please find them on Pages 14-15 of this response letter.*

13.	For visualization purposes, it is worth showing the actual MSE comparison of those summary statistics for 8 simulators rather than simply plotting the distribution.

*Response:*

*We could not do this because for the simulators that do not preserve genes, the MSE cannot be calculated. Here is the quote from the text (Page 6):*

*“Note that we cannot compare these statistics' values for simulators that do not preserve genes because the “genes” in those simulators' synthetic data cannot be matched to any genes in real data.”*

14.	Page 7, last paragraph: " we still benchmark scDesign2 against ZINB-WaVE and SPARSim, the two simulaors" Typo: 'simulaors' → 'simulators'

*Response:*

*Thanks. We have corrected the typo.*

*Reference*

*1.	Avramidis AN, Channouf N, L’Ecuyer P. Efficient Correlation Matching for Fitting Discrete Multivariate Distributions with Arbitrary Marginals and Normal-Copula Dependence. INFORMS J Comput. INFORMS; 2009;21:88–106.*

*2.	Lebrun R, Dutfoy A. An innovating analysis of the Nataf transformation from the copula viewpoint. Probab Eng Mech. 2009;24:312–20.*

*3.	Liu B, Li C, Li Z, Wang D, Ren X, Zhang Z. An entropy-based metric for assessing the purity of single cell populations. Nat Commun. 2020;11:3155.*

*4.	Stuart T, Butler A, Hoffman P, Hafemeister C, Papalexi E, Mauck WM 3rd, et al. Comprehensive Integration of Single-Cell Data. Cell. 2019;177:1888–902.e21.*

*5.	Blondel VD, Guillaume J-L, Lambiotte R, Lefebvre E. Fast unfolding of communities in large networks. J Stat Mech. IOP Publishing; 2008;2008:P10008.*

*6.	MacQueen, J, author. Some methods for classification and analysis of multivariate observations. Proceedings of the Fifth Berkeley Symposium on Mathematical Statistics and Probability, Volume 1: Statistics. University of California Press; 1967. p. 281–97.*

*7.	Kiselev VY, Kirschner K, Schaub MT, Andrews T, Yiu A, Chandra T, et al. SC3: consensus clustering of single-cell RNA-seq data. Nat Methods. 2017;14:483–6.*

*8.	Bien J, Tibshirani RJ. Sparse estimation of a covariance matrix. Biometrika. [Oxford University Press, Biometrika Trust]; 2011;98:807–20.*

*9.	Korsunsky I, Millard N, Fan J, Slowikowski K, Zhang F, Wei K, et al. Fast, sensitive and accurate integration of single-cell data with Harmony. Nat Methods. Nature Publishing Group; 2019;16:1289–96.*

*9.	Ding J, Adiconis X, Simmons SK, Kowalczyk MS, Hession CC, Marjanovic ND, et al. Systematic comparison of single-cell and single-nucleus RNA-sequencing methods. Nat Biotechnol. Nature Publishing Group; 2020;38:737–46.*

*10.	Jiang R, Sun T, Song D, Li JJ. Zeros in scRNA-seq data: good or bad? How to embrace or tackle zeros in scRNA-seq data analysis? [Internet]. bioRxiv. 2020 [cited 2021 Apr 3]. p. 2020.12.28.424633. Available from: http://dx.doi.org/10.1101/2020.12.28.424633*

**Second round of review**

**Reviewer 1**

The authors have addressed all my previous questions. One minor spelling issue: "principle component analysis" should be spelled as "principal component analysis" throughout the paper (e.g., Fig. 4b legend).

**Reviewer 2**

The authors have addressed all of my concerns, and improved the manuscript substantially. I also appreciate the authors' experiments and candid remarks re: cross platform simulation, and look forward to reading future work from them on this.
